# From chemical characterization to functional validation: Screening of anti-hyperglycemic components and their action mechanisms in differentially fermented teas

**DOI:** 10.1016/j.fochx.2026.103721

**Published:** 2026-03-06

**Authors:** Guangneng Li, Jianyong Zhang, Ying Gao, Hongchun Cui, Debao Niu, Junfeng Yin

**Affiliations:** a Tea Research Institute Chinese Academy of Agricultural Sciences, National Key Laboratory for Tea Plant Germplasm Innovation and Resource Utilization, National Engineering Research Center for Tea Processing, Hangzhou, China; b College of Light Industry and Food Engineering, Guangxi University, Nanning 530003, China; c Tea Research Institute, Hangzhou Academy of Agricultural Sciences, Hangzhou 310024, China

**Keywords:** *Jinxuan*, Fermented and non-fermented tea, UHPLC-Q-Exactive /MS, theasinensinA, *Db/db* mice

## Abstract

Single-source *Jinxuan (Camellia sinensis)* tea leaves were processed into teas with varying fermentation degrees to minimize genetic and environmental interference. Non-volatile component differences between fermented and non-fermented teas were identified, with fermented teas showing reduced tea polyphenols and soluble sugars but elevated theaflavins and theasinensin A(TSA). Fermented teas exhibited stronger in vitro digestive enzyme inhibition, particularly α-glucosidase (α-Glu), with TSA (*R* = 0.97) and theaflavin-3-gallate (*R* = 0.93) showing the strongest correlations (*p* < 0.001). In vivo, TSA's hypoglycemic effect in diabetic mice was mediated by enhanced insulin secretion, linked to lipid, amino acid, and fatty acid metabolism. These findings elucidate fermented tea's superior digestive enzyme inhibition and provide a foundation for optimizing hypoglycemic effects through processing.

## Highlights

• Fermented teas show stronger digestive enzyme inhibition in vitro.
• Theasinensin A and theaflavins positively correlate with enzyme inhibition.
• Theasinensin A lowers postprandial glucose via enhanced insulin secretion.
• Theasinensin A regulates blood glucose and links to lipid/amino acid metabolism.

## Graphical abstract

**Figure:**
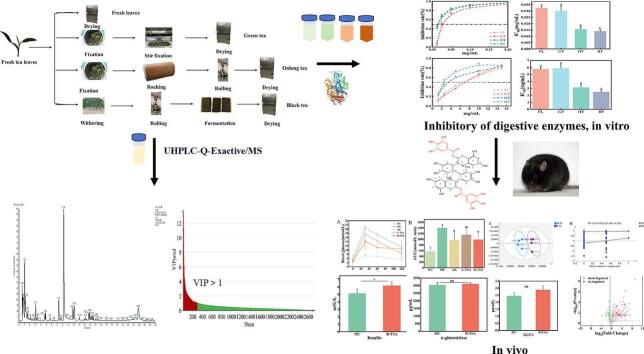

## 1 Introduction

Diabetes, a complex metabolic disorder, poses a significant global public health challenge due to its widespread prevalence. Beyond pharmacological treatment, dietary intervention—particularly through the modulation of postprandial blood glucose using functional food components—has emerged as a crucial strategy for the prevention and management of type 2 diabetes (T2D). Among the numerous potential dietary factors, tea has attracted considerable attention due to its rich array of bioactive compounds. Epidemiological and intervention studies have consistently indicated that tea consumption, particularly of black tea, is associated with a reduced risk of T2D and lower postprandial blood glucose levels(Aloo et al., 2024; Ma et al., 2023). This benefit is widely attributed to specific active components transformed during the tea fermentation process (e.g., theaflavins, theasinensins), which can directly modulate postprandial glucose by inhibiting the activities of intestinal α-amylase and α-glucosidase, thereby reducing the digestion of carbohydrates into glucose(G. Li et al., 2025; Zhao et al., 2024).

However, significant controversy remains regarding “which type of tea is more effective for lowering blood glucose,” primarily due to critical limitations in existing studies. The first is the heterogeneity of source materials. Most comparative studies use commercially available teas, which vary in cultivar, origin, and processing techniques. This leads to substantial differences in chemical composition, confounding the results and preventing clear attribution of effects to the core variable: the fermentation process. Secondly, there is a lack of component-activity correlation. Although it is known that fermented tea (e.g., black tea) extracts exhibit potent digestive enzyme inhibitory activity in vitro (Liu et al., 2021; Z. Wang et al., 2024), research has largely remained at the extract level. It has failed to systematically elucidate the relationship between the dynamic changes of key active components (e.g., catechins, theaflavins, theabrownins) and their hypoglycemic potency. Finally, there is an inadequate elucidation of in vivo mechanisms. In vitro enzyme inhibition models cannot fully replicate the complex physiological environment in vivo. The absorption, metabolism, and impacts of key active components on systemic pathways regulating blood glucose—such as those involving incretins (e.g., glucagon-like peptide-1(GLP-1))—still lack systematic validation.

Based on this, a single batch of fresh tea leaves *(Jinxuan)* was processed using standardized methods to produce non-fermented teas (fresh leaves, green tea) and fermented teas (oolong tea, black tea), thereby fundamentally eliminating confounding factors related to cultivar and geographical origin. Targeted quantification (high-performance liquid chromatography (HPLC) analysis of catechins and theaflavins) was integrated with untargeted metabolomics ultrahigh-performance liquid chromatography-quadrupole/electrostatic field orbitrap mass spectrometer (UHPLC-Q-Exactive/MS) to comprehensively reveal the dynamic evolution of chemical components with fermentation degree. This chemical profile was then correlated with in vivo hypoglycemic activity to precisely screen candidate active compounds. The screened core compounds were administered via gavage to *db/db* mice. Their in vivo hypoglycemic efficacy was evaluated from multiple perspectives using the oral glucose tolerance test (OGTT), measurements of serum insulin and GLP-1 levels, serum metabolomics, and pharmacokinetic analysis, thereby providing preliminary elucidation of their mechanisms of action. This study aims, through the above design and under conditions of strictly controlled raw materials, to establish the relationship between tea fermentation degree, specific chemical components, and hypoglycemic activity, thereby providing a scientific foundation for developing tea products with precise blood glucose management functions.

## 2 Materials and methods

### 2.1 Materials

In July 2023, *Jinxuan* fresh leaves (one bud and two leaves) were harvested from the experimental base located in Shengzhou, Zhejiang Province. The total weight of fresh leaves, amounting to 26 kg, was divided into four parts: fresh leaf drying samples (1 kg), green tea (5 kg), oolong tea (5 kg), and black tea (15 kg). Fresh leaves were directly fixed using hot air at 120 °C for 30 min and subsequently baked at 85 °C until completely dry to obtain fresh leaf drying samples. The collected fresh leaves were withered indoors to a moisture content of 71.07% (7 h, 28 °C), followed by fixation using an automatic flat tea stir-frying machine (220 °C, 10 min). The leaves were then dried using a tea aroma extractor machine (model 6CHT-16, Zhejiang Zhufeng Machinery Co.) to a moisture content of 1.17% (2 h, 85 °C) to obtain fried green tea. The fresh leaves underwent outdoor withering (34 °C, 0.5 h), reaching a moisture content of 71.01% (Rapid Halogen Moisture Detector (Xinchang Junyi Machinery Co.). They were then processed using a 6CWY-60-type wave green machine (1 h, 30 rpm/min; spreading for 30 min; 1 h, 50 rpm/min, Fujian Jiayou Tea Machine Co.) and finally baked for 2 h to complete drying, resulting in oolong tea samples. Black tea was prepared using a tea rolling machine (model 6CRM-25, Zhejiang Chunjiang Tea Machine Co.) with the following steps: light rolling (10 min), heavy rolling (20 min) and light rolling (5 min). The leaves were then fermented in an intelligent artificial climate chamber (model PRX—500D, Ningbo Plante Instrument Co.) at 90% humidity and 20 °C for 5 h, followed by baking to complete dryness to obtain black tea samples (Fig. S1).

### 2.2 Main chemical reagents

Acarbose was obtained from Shanghai Dibai Biotechnology Co., Ltd. *p*-Nitrophenyl-α-D-glucopyranoside (p-NPG), α-Glu (CAS 9001-42-7), and α-amylase (derived from *Bacillus subtilis*, CAS 9000-90-2) were purchased from Shanghai Yuanye Biotechnology Co., Ltd. Analytically pure tapioca starch was procured from Baiji (Hubei) Biotechnology Co., Ltd. DNS reagents were acquired from Beijing Solebaum Technology Co., Ltd. The α-Glu ELISA kit was obtained from Hangzhou Xincheng Bio-technology Co., Ltd. A blood glucose meter and test strips (Sinocare/Sanno) as well as soluble starch (for gavage) were purchased from Nanjing Chemical Reagent Co., Ltd. Theasinensin A(TSA) was prepared according to previous literature with a purity of 89.28%(J. Zhang et al., 2023). All other reagents and chemicals used were of chromatographic or analytical grade.

### 2.3 Methods

#### 2.3.1 Tea infusion extraction

The resulting tea was ground into a homogeneous powder using a pulverizing grinder (IKA, Germany, 20000 rpm/min, 20 s), and 0.2 g of the tea sample was accurately weighed into a test tube. The sample was then mixed with 10 mL of boiling water and extracted in a water bath maintained at 100 °C. The samples were incubated for 10 min with intermittent shaking every 5 min. Afterwards, the samples were rapidly cooled and centrifuged for 10 min at 8000 rpm/min using a centrifuge (model 5810R, Eppendorf, Hamburg, Germany). The resulting supernatant was filtered through a 0.45 μm membrane filter. Three replicates were performed for each sample.

#### 2.3.2 Determination of tea polyphenols and tea pigments (TFs-TRs-TBs) in tea samples

The determination of tea polyphenol content was conducted in accordance with a previously published method, which was aligned with the National Standard of China GB/T 8313–2018(Y. Gao et al., 2020). Following slight modification, One mL of tea infusion obtained from subsection 2.3.1 was transferred into a one hundred mL volumetric flask, where it was thoroughly shaken and diluted to the marked volume. One mL aliquot was then mixed with five mL of 10% Folin-Ciocalteu reagent. The mixture was vigorously shaken and allowed to stand for 3–8 min. Subsequently, four mL of 7.5% sodium carbonate solution was added, followed by thorough shaking. The solution was permitted to remain stationary for 60 min to detect absorbance, serving as a blank control, with pure water used to measure absorbance at 765 nm (Ultraviolet spectrophotometer 2000, Unico Instrument (Shanghai) Co.)

Tea pigments (TFs, TRs, and TBs) were determined using the method described by Hua et al.(G. Li, Zhang, Cui, Gao, et al., 2024). A 3.00 g sample of ground tea was accurately weighed and dissolved in 125 mL of boiling water. Extraction was performed for 10 min with periodic stirring every 5 min. The tea extract was obtained by filtration while the solution remained hot. One hundred mL split funnel was filled with 25 mL of ethyl acetate and 25 mL of tea extract, and the mixture was shaken for 5 min to perform extraction. The two layers were separated, and the aqueous layer (lower layer) was removed. The ethyl acetate layer (upper layer) was collected and stored. Two mL of the ethyl acetate layer was diluted to 25 mL with 95% ethanol to prepare solution A. Two mL of the aqueous layer was mixed with 2 mL of saturated oxalic acid solution and 6 mL of distilled water, then diluted to 25 mL with 95% ethanol to prepare solution B. Fifteen mL of the ethyl acetate layer and 15 mL of 2.5% sodium bicarbonate solution were combined in a 50 mL dispersion funnel and shaken vigorously for 30 s. After standing and separating, 4 mL of the upper layer was diluted to 25 mL with 95% ethanol to prepare solution C. Finally, 15 mL of tea extract and 15 mL of n-butanol were mixed in a 50 mL dispersion funnel, shaken for 3 min, and after standing for layering, 2 mL of the aqueous layer was taken into a 25 mL volumetric flask, and 2 mL of saturated oxalic acid solution and 6 mL of distilled water were sequentially added, and finally, 95% ethanol was added to get solution D. Absorbance measurements at 380 nm yielded values for EA, EB, EC, and ED. The percentages of TFs, thearubigins(TRs), and theabrownins(TBs) were calculated using the formulas: TFs (%) = 2.25 × EC; TRs (%) = 7.06 × (2 × EA + 2 × EB − EC − 2 × ED); and TBs (%) = 2 × ED × 7.06.

#### 2.3.3 Detection of soluble sugars in samples

The determination of soluble sugars was performed using the anthrone‑sulfuric acid colorimetric method, which mirrors the approach previously described by the subject (Y. Gao et al., 2022). One mL of the sample solution was mixed with 4 mL of anthrone‑sulfuric acid (2 mg/mL), and the mixture was incubated in a water bath at 100 °C for 10 min. After cooling to room temperature, the absorbance at 620 nm was measured.

#### 2.3.4 Analysis of catechins and theaflavins in tea by ultra-high liquid chromatography

The method described by Li et al.(G. Li, Zhang, Cui, Gao, et al., 2024) was followed. A 5C18-AR-II chromatographic column (4.6 mm × 250 mm) was utilized. The injection volume was set at 10 μL, and the detection wavelength was fixed at 280 nm. The flow rate was maintained at 0.8 mL/min, and the column temperature was controlled at 35 °C. The mobile phases were composed of phase A (50 mmol/L phosphoric acid) and phase B (100% acetonitrile). The elution gradients were programmed as follows: from 0 to 39 min, phase A was decreased from 96% to 70%; from 39 to 54 min, phase A was further decreased from 70% to 25%; and from 54 to 55 min, phase A was increased from 25% to 96%.

#### 2.3.5 Untargeted metabolomics analysis of teas with varying fermentation degrees

Metabolomic analysis of tea samples was performed using UHPLC-Q-Exactive/MS (Thermo Fisher, San Jose, CA, USA). To ensure instrument stability and data accuracy, quality control (QC) samples were prepared by mixing aliquots of different tea infusion extracts(J. Gao et al., 2023). QC samples were analyzed once for every three tea samples, and a blank sample was injected following each QC run to minimize residual effects during sample analysis. The UHPLC-Q-Exactive/MS system was equipped with a T3 column (100 mm × 2.1 mm, 1.8 μm; Waters, Ireland), and the column temperature was maintained at 40 °C with a flow rate of 0.4 mL/min. The injection volume was set at 3 μL. The mobile phases consisted of phase A (0.1% formic acid in water) and phase B (0.1% formic acid in acetonitrile). The linear gradient elution program was set as follows: 0 min, 2% B; 0.5 min, 2% B; 10 min, 15% B; 18 min, 40% B; 20 min, 90% B; 20.9 min, 90% B; 21 min, 2% B; and 25 min, 2% B. The mass spectrometer equipped with an electrospray ionization (ESI) source was operated in positive ionization mode. The capillary voltage and temperature were set at 3500 V and 300 °C, respectively; the drying gas flow rate and temperature were maintained at 10 L/min and 325 °C, respectively; and a mass scan range of *m*/*z* 100–1000 was applied(J. Gao et al., 2023).

#### 2.3.6 A preliminary study on the inhibitory activity of digestive enzymes in teas with different degrees of fermentation

##### 2.3.6.1 α-glucosidase inhibition assay

Based on previous literature, the hypoglycemic effect of tea infusion was evaluated through in vitro α-Glu inhibition assays(G. Li, Zhang, Cui, Gao, et al., 2024). Tea infusions at different concentrations (50 μL; 0.20, 0.10, 0.05, 0.025, and 0.0125 mg/mL) was incubated with α-Glu (50 μL, 1 U/mL) in phosphate buffer (0.1 M, pH 6.8) at 37 °C for 10 min. Subsequently, *p*-nitrophenyl-α-D-glucopyranoside (p-NPG; 100 μL, 5 mM) was added and allowed to react for 15 min, followed by the immediate addition of sodium carbonate (Na_2_CO_3_; 300 μL, 1 M). The absorbance at 405 nm was then measured using a multifunctional microplate reader (CYTATION1, BioTek, Burlington, Vermont, USA). Sample and blank controls were included, and acarbose was used as the positive control. The inhibitory activity of α-Glu was calculated using the following formula:

$$\alpha -\mathrm{Glu}\;\text{inhibition}\left(\%\right)=\left(1-\left(A1-A2\right)/\left(A3-A4\right)\right)\times 100\%$$

Where, A1 and A2 denote the absorbance values of the sample group and the sample blank group, respectively; A3 and A4 denote the absorbance values of the blank group and the blank control, respectively, and the results are expressed as IC_50_ values.

#### 2.3.7 .2 α-amylase inhibition assay

The α-amylase inhibitory capacity of tea samples was determined using the methodology described by Li et al.(G. Li, Zhang, Cui, Gao, et al., 2024). After extraction, tea infusions were mixed with α-amylase (0.5 U/mL; PBS, 0.1 M, pH 6.9, 6.6 mm NaCl) at varying concentrations (0.2 mL; 15, 10, 5, 2.5, and 1.25 mg/mL) in 2 mL centrifuge tubes and incubated at 25 °C for 10 min. Tapioca starch (10 mg/mL, 0.2 mL) was subsequently added to the mixture, which was further incubated for an additional 10 min. The procedure was completed by heating 0.4 mL of DNS reagent at 100 °C for 10 min. Following cooling, 0.1 mL of the reaction solution was transferred to a 96-well enzyme-labeled plate and assayed at 540 nm using an enzyme marker to evaluate α-amylase inhibition.

$$\alpha -\text{amylase inhibition}\left(\%\right)=\left(1-\left(A1-A2\right)/\left(A3-A4\right)\right)\times 100\%$$

Where, A1 and A2 denote the absorbance values of the sample group and the sample blank group, respectively; A3 and A4 denote the absorbance values of the blank group and the blank control, respectively, and the results are expressed as IC_50_ values.

#### 2.3.8 Dose information determination

The primary objective of this study was to evaluate the effects of TSA on postprandial glycaemic control in a db/db mouse model of T2D. The secondary objectives were to assess its impact on serum insulin, GLP-1, and intestinal α-glucosidase activity. Human limited consumption of epigallocatechin gallate (EGCG) is 300 mg/day according to the National Health Commission of the People's Republic of China (http://www.nhc.gov.cn/wjw/index.shtml, Circular No.17 of 2010), then the equivalent mice dosage of EGCG can be calculated as 61.5 mg/day by the formula in FDA guidance(Food Drug Administration, 2005). TSA is a dimer of EGCG, hence the mole of 100 mg is equivalent with that of 50 mg EGCG. Thus, low (50 mg /kg), and high (100 mg/kg) dosages of TSA were set for the treatments in the present study. In addition, acarbose was set as a control (50 mg/kg). The experimental unit was the individual animal.

#### 2.3.9 Laboratory animal feeding and grouping

Twenty male db/db mice (44.99 ± 2.56 g) and five db/m mice (24.92 ± 0.65 g) at 8 weeks of age, sourced from Shanghai Slarc Laboratory Animal Co., Ltd. (Shanghai, China), were housed in the Animal Experiment Centre of Zhejiang Chinese Medical University (ethical approval code: SYXK (ZHE) 2021–0012). Mice were housed in groups of 4–5 per individually ventilated cage (IVC) with corn cob bedding. Nesting material and a plastic shelter were provided in each cage as environmental enrichment.

The temperature and humidity in the sterile barrier environment were maintained at 24 ± 2 °C and 50% - 70%, respectively, with intermittent illumination of 12 h:12 h day/night. Throughout the study, all mice had ad libitum access to a standard laboratory rodent diet (SPF Rodent Maintenance Diet, Animal Experiment Centre of Zhejiang Chinese Medical University, Hangzhou, China) and autoclaved tap water.

After acclimatization, twenty db/db mice were fed ad libitum for three days with the aforementioned standard diet, and they were randomly divided into four experimental groups of five mice. The model control (MC) group received gavage with starch (2 g/kg body weight) and equal volumes of water (0.2 mL). The acarbose high-dose group (AK, 50 mg/kg) received gavage with starch (2 g/kg body weight). The TSA low/high-dose group (50 mg/kg/100 mg/kg;L-TSA/H-TSA) received gavage with starch (2 g/kg body weight). The 5 db/m mice in the normal control group (NC) received gavage with starch (2 g/kg body weight) and equal volumes of water (0.2 mL). All mice were gavaged in the order of starch first, then reagent, (Table S3). Animal care staff were not blinded to group allocation due to the nature of the gavage treatments. However, the researchers performing the blood glucose measurements, sample assays (insulin, GLP-1, α-glucosidase), and data analysis were blinded to the group identity of the samples.

#### 2.3.10 Oral glucose tolerance test

After three days of acclimatization feeding, fasting for 12 h and measuring fasting blood glucose, each group of mice was sequentially infused with starch (2 g/kg body weight). The primary outcome measure for this test was the area under the blood glucose-time curve from 0 to 120 min (AUC₀–₁₂₀). Secondary measures included individual time-point glucose values.

Real-time blood glucose values were measured in each group after 0, 30 min, 60 min, and 120 min, respectively (Blood glucose meter and accompanying test strips, Sinocare), and the area under the blood glucose-time curve (AUC) was calculated. Blood was collected at 180 min and 300 min for the H-TSA group. At the conclusion of the experiment, euthanasia was performed on the mice. This procedure was strictly conducted in accordance with the Chinese National Standard GB/T 39760–2021 *Laboratory animal—Guidelines for euthanasia*(State Administration for Market Regulation & Standardization Administration of China, 2021). The carbon dioxide inhalation method was employed. The specific steps were as follows: mice were placed into a clean, airtight euthanasia chamber. No gas was pre-charged into the chamber prior to introducing the animals. Subsequently, 100% carbon dioxide was introduced at a flow rate displacing 20%–30% of the chamber volume per minute. The gas flow was terminated once all animals exhibited cessation of movement, absence of breathing, and fully dilated pupils. The animals were maintained within the gas-filled chamber for an additional 2–3 min. Death was then conclusively confirmed by assessing the absence of vital signs, including lack of respiration, absence of heartbeat, and loss of corneal reflex.

#### 2.3.11 Animal welfare and ethical considerations

The following specific measures were implemented throughout the study to ensure animal welfare and scientific validity. 1. A 12-h fasting period was implemented prior to the oral starch tolerance test to ensure a standardized post-absorptive state and to allow for an accurate assessment of postprandial glucose metabolism. This duration is standard practice in diabetic rodent models, including db/db mice, as it reliably establishes baseline glycaemia while minimizing the risk of severe hypoglycemia. 2. All blood samples were collected from the retro-orbital plexus by trained personnel to minimize pain and distress. The sampling details were as follows: blood samples at 180 min (∼100 μL) and 300 min (∼100 μL) were collected from the H-TSA group (*n* = 5). For a separate cohort, blood samples at 0 min (∼100 μL) and 30 min (∼900 μL) were collected prior to euthanasia by trained personnel (cervical dislocation) (*n* = 4 per time point; tables S3 and S4 for details). The 0- and 30-min samples were from the MC and H-TSA groups described in 2.3.11, 2.3.12, while the 180- and 300-min samples were from the H-TSA subgroup in section 2.3.8. This two-cohort design for H-TSA metabolite analysis was employed to avoid excessive blood volume withdrawal from any single animal. Following centrifugation, serum was collected and pooled accordingly. For the serum metabolomics analysis of H-TSA, a 60 μL aliquot of serum was used per time point. 3. All animals were monitored at least twice daily by trained personnel for general health indicators, including body condition, posture, activity, and food and water intake. Predefined humane endpoints, requiring immediate euthanasia, included: 1) >20% body weight loss within 48 h; 2) severe lethargy, immobility, or inability to reach food or water; 3) signs of severe dehydration or hypothermia; and 4) any self-inflicted injury or significant ulceration. No animals reached these endpoints during the study. 4. We acknowledge that the experimental procedures, including gavage, fasting, blood sampling, and terminal procedures, imposed a cumulative burden on the animals. This burden was actively mitigated by employing skilled personnel for all procedures, minimizing sample volumes, providing environmental enrichment, and maintaining optimal housing conditions. The study protocol was designed to use the minimum number of animals necessary, in strict adherence to the 3Rs principles (Replacement, Reduction, and Refinement).

#### 2.3.12 Statistical methods

All data are presented as mean ± standard deviation (SD). For comparisons of the primary outcome (AUC₀–₁₂₀) and other parameters across the five main groups (NC, MC, AK, L-TSA, H-TSA), one-way analysis of variance (ANOVA) was performed, followed by Tukey's post-hoc test for multiple comparisons. For the two-group comparison in section 2.3.10, an unpaired two-tailed Student's *t*-test was used. Statistical significance was set at *p* < 0.05. All analyses were conducted using Origin 2021 software.

#### 2.3.13 Determination of insulin level and GLP-1 level in serum and α-glucosidase level in intestinal contents

Another 8 *db/db* mice were randomly divided into two groups of four mice each (Table S4). The MC group received gavage with starch (2 g/kg body weight) and equal volumes of water. The H-TSA group (100 mg/kg) received gavage with starch (2 g/kg body weight). After fasting for 12 h in each group, before administering gavage of starch (2 g/kg body weight), blood was collected from the corners of the eyes at 0 min, and blood samples were collected again after 30 min. Cervical dislocation was utilized as the euthanasia method, and the contents of the small intestines (12–15 cm below the stomach) were collected. The blood was centrifuged at 4000 rpm for 10 min at 4 °C to obtain serum. Postprandial blood glucose levels of mice were measured using the kit. The collected intestinal contents were added to 200 μL of phosphate buffer (0.01 M, pH 6.8), mixed by vortexing for 2 min, and then centrifuged at 12,000 rpm at 4 °C to obtain 10 μL of sample. 40 μL of sample dilution was added to measure α-glucosidase content according to the kit method.

#### 2.3.14 Analysis of in vivo metabolism of TSA in *db/db* mice

Blood was collected from MC and H-TSA mice at 0-min, 30-min, 180-min, and 300-min time points after oral administration of H-TSA. The in vivo metabolism of H-TSA was detected at each time point. The serum samples (60 μL) were thoroughly mixed with 200 μL of methanol and centrifuged at 12,000 rpm for 10 min at 4 °C in a cryogenic centrifuge. The extracted supernatant was transferred to a new 2 mL centrifuge tube. The supernatant was blown with nitrogen until it reached a viscous consistency, and 100 μL 80% aqueous methanol was added to re-solubilize the sample. The supernatant was passed through a 0.22 μm filter membrane, and the filtrate was collected for testing. The method was the same as that described in Section 2.3.5.

### 2.4 Statistical analysis of data

The experiment was repeated three times for each group and the results were expressed as mean ± standard deviation (SD). Statistical significance between the two groups was analyzed using an independent samples *t*-test. Results were analyzed by one-way analysis of variance (ANOVA) and Duncan post-hoc test, and IC_50_ was calculated using SPSS 26.0 (SPSS Inc., Chicago, IL, USA) software and was considered statistically significant at *p* < 0.05. Origin 2021 software (Origin Lab, Northampton, MA, USA) was used to draw graphs.

## 3 Results and discussion

### 3.1 Physicochemical composition of different tea types

Tea polyphenols, which constituted the primary non-volatile components of tea, accounted for 18–36% of the dry weight of tea leaves and primarily included catechins, flavonols, anthocyanins, and phenolic acids. As shown in Table 1, a significant decrease (*p* < 0.05) in tea polyphenol content was observed with increasing fermentation degree. The content in fresh leaves (14.93 ± 0.58%) was significantly higher than that in oolong tea (8.94 ± 0.78%) and black tea (9.24 ± 0.29%).

**Table 1:** Major components of tea with different degrees of fermentation (mg/g).

Samples	Tea polyphenols	Total soluble sugars	TFs	TRs	TBs	Total tea pigment
FL	14.93 ± 0.58a	8.94 ± 0.30a	/	/	/	/
GT	14.04 ± 0.59a	8.66 ± 0.17a	/	/	/	/
OT	8.94 ± 0.78b	8.04 ± 0.24b	0.30 ± 0.03a	4.33 ± 0.06b	6.54 ± 0.11b	11.17 ± 0.20a
BT	9.24 ± 0.29b	8.24 ± 0.36b	0.34 ± 0.00a	7.14 ± 0.23a	7.36 ± 0.13a	14.84 ± 0.11b

Note: FL, GT, OT, and BT denote fresh leaves, green tea, oolong tea, and black tea, respectively. Data are expressed as mean ± standard deviation (*n* = 3). Different lowercase letters in the same column indicate significant differences at the *p <* 0.05 level. ‘/’ represents the inappropriateness of the systematic method for pigment measurement.

Soluble sugars in tea, which primarily consisted of monosaccharides, disaccharides, and a small amount of oligosaccharides, were identified as the main source of sweetness in tea infusion and were positively correlated with tea flavor(L. Zhang et al., 2020). As shown in Table 1, the accumulation of soluble sugars was favored during green tea processing, and no significant difference was observed compared to fresh leaves (*p >* 0.05). As the degree of fermentation increased, a significant decrease in soluble sugar content was observed, which was attributed to the formation of precipitates resulting from the combination of sugars and oxidized tea polyphenols with numerous polyphenol hydroxyl groups (Huang et al., 2015). Additionally, the differences in pigment contents between fermented teas were further analyzed. No significant difference in total TFs was detected using a systematic method (*p* > 0.05). However, the contents of TRs and TBs were significantly higher in black tea compared to oolong tea (*p* < 0.05), suggesting that further fermentation promoted the formation of tea pigments.

### 3.2 Catechins and theaflavins in different tea types

Catechins, which were identified as the primary active components in tea, were further classified into galloylated and non-galloylated catechins based on their structural characteristics.

As shown in Table 2, the contents of eight catechin fractions (gallocatechin (GC), epigallocatechin (EGC), catechin (C), epicatechin (EC), EGCG, gallocatechin gallate (GCG), epicatechin gallate (ECG), and catechin gallate (CG)) and four theaflavin fractions (theaflavin (TF), theaflavin-3-gallate (TF3G), Theaflavin-3′-gallate (TF3’G), and theaflavin-3,3′-digallate (TFDG)) were quantified. The total catechin (TCs) content in fresh leaves was measured at 105.46 mg/g, and a decreasing trend was observed after processing into different tea types. A significant decrease in the total catechin content was observed with increasing fermentation degree. The TCs content was determined to be 83.34 mg/g in green tea, 32.06 mg/g in oolong tea, and 21.93 mg/g in black tea, which was significantly lower than that in other tea types. This suggested that the rolling and fermentation stages during black tea processing significantly influenced catechin content changes.

**Table 2:** Analysis of catechins theaflavins of teas with different degrees of fermentation (mg/g).

	FL	GT	OT	BT
GC	15.69 ± 0.95a	10.05 ± 0.68b	5.32 ± 0.26c	5.66 ± 0.12c
EGC	1.61 ± 0.10a	1.78 ± 0.25a	0.21 ± 0.04b	0.29 ± 0.01b
C	2.59 ± 0.23a	2.57 ± 0.04a	1.09 ± 0.24b	0.13 ± 0.00c
EC	9.89 ± 0.40a	9.05 ± 0.26b	2.49 ± 0.24c	0.93 ± 0.01d
EGCG	59.97 ± 1.57a	47.37 ± 1.87b	15.80 ± 0.74c	7.74 ± 0.56d
GCG	0.33 ± 0.02a	0.84 ± 0.09b	0.49 ± 0.04c	0.03 ± 0.00d
ECG	13.69 ± 0.32a	10.55 ± 0.37b	5.35 ± 0.88c	5.01 ± 0.50c
CG	1.71 ± 0.09a	1.13 ± 0.06b	1.32 ± 0.12c	2.14 ± 0.07d
Galloylated catechins	75.69 ± 1.99a	59.89 ± 2.40b	22.95 ± 1.79c	14.92 ± 1.13d
Non-galloylated catechins	29.77 ± 1.68a	23.45 ± 1.22b	9.10 ± 0.77c	7.01 ± 0.15c
TCs	105.46 ± 3.67a	83.34 ± 3.62b	32.06 ± 2.56c	21.93 ± 1.28d
TF	0.26 ± 0.02c	0.32 ± 0.01c	1.05 ± 0.06b	1.23 ± 0.04a
TF3G	0.04 ± 0.00c	0.07 ± 0.01c	0.68 ± 0.06b	1.02 ± 0.03a
TF3’G + TFDG	0.10 ± 0.01c	0.02 ± 0.00c	1.45 ± 0.14a	0.89 ± 0.03b
TFs	0.40 ± 0.02b	0.41 ± 0.02b	3.18 ± 0.26a	3.15 ± 0.11a

Note: Different letters in the same line indicate significant differences (*p* < 0.05).

The catechin fractions in fresh leaves were ranked in descending order as EGCG, GC, ECG, EC, C, CG, EGC, and GCG. EGCG was found to account for more than half of the total catechins in fresh leaves and green tea, while its proportion was reduced to less than half in oolong and black tea. A significant decreasing trend (*p* < 0.05) was observed in all catechin fractions in both non-fermented (fresh leaves and green tea) and fermented (oolong tea and black tea) teas. The content of alloylated catechins (EGCG, ECG, CG, and GCG) decreased significantly from 75.69 mg/g in fresh leaves to 59.89 mg/g in green tea, 22.95 mg/g in oolong tea, and 14.92 mg/g in black tea. Similarly, the content of non- alloylated catechins (EGC, EC, C, and GC) also exhibited a significant decrease during the processing of fresh leaves into green tea, oolong tea, and black tea. Alloylated catechins accounted for more than half of the non- alloylated catechins in teas with varying degrees of fermentation, indicating that the alloylated catechins content was primarily determined by the EGCG content. For both alloylated and non-alloylated catechins, the content in non-fermented teas was significantly higher than that in fermented teas. This suggested that during the processing of oolong tea and black tea, the rocking process in oolong tea and the rolling and fermentation processes in black tea promoted the oxidation of catechins, particularly the high-content alloylated catechins.

During the fermentation process of tea, tea polyphenols (predominantly catechins) were initially converted into quinones through enzymatic oxidation reactions mediated by polyphenol oxidase and peroxidase. Additionally, catechin oxidation to quinones was also observed to occur via an autocatalytic reaction pathway. Subsequently, these catechins were further oxidized and polymerized, resulting in the formation of TFs and TRs(Zhao et al., 2024). Therefore, the TFs content was comparatively analyzed across different tea categories. Significantly higher concentrations were detected in fermented teas, with oolong (3.18 mg/g) and black tea (3.15 mg/g) demonstrating markedly elevated levels compared to non-fermented counterparts. Trace amounts of TFs were identified in both green tea and fresh leaves, indicating that enzymatic oxidative processes were initiated immediately upon leaf harvesting. The combined effects of hydric stress and mechanical injury induced progressive oxidation of cellular constituents in fresh leaves, culminating in the gradual accumulation of TFs and related compounds (Li et al., 2020; Tanaka et al., 2010).

### 3.3 LC-MS-based non-targeted metabolomics analysis of different teas

#### 3.3.1 Overall metabolite distribution in different tea types

**Fig. 1:**
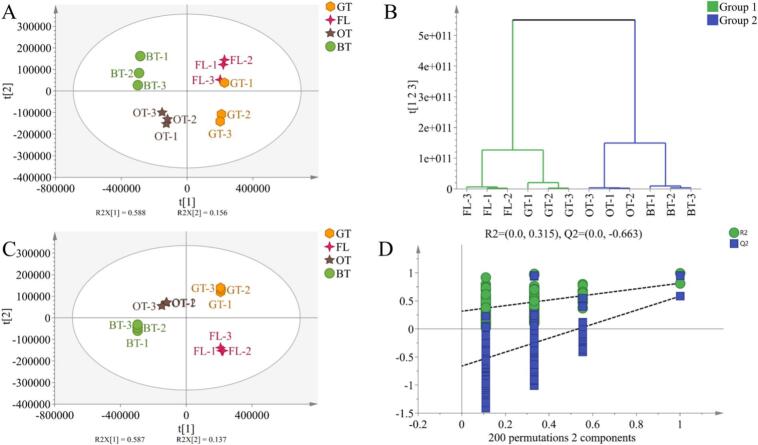
Tea PCA(A), HCA(B), OPLA-DA (C)with different degrees of fermentation, 200 times validation plot analysis(D). Hierarchical cluster analysis was performed using Euclidean distance as the distance metric.

The comprehensive metabolic alterations in four tea groups with varying fermentation degrees were investigated using UHPLC-Q-Exactive/MS integrated with high-coverage untargeted metabolomic profiling. To elucidate phenotypic differences among samples, principal component analysis (PCA) was performed on 2741 feature peaks in positive ion mode. In the positive ion mode analysis, score plot of the PCA model based on metabolite profiles. The first two principal components (PC1 and PC2) explained 58.8% and 15.6% of the total variance, respectively, with a cumulative explained variance of 74.4% (Fig. 1A). All experimental replicates fell within the 95% confidence ellipses with no detectable outliers requiring exclusion. PCA score plots revealed group-specific clustering patterns corresponding to biological replicates, demonstrating significant metabolic disparities among tea groups with differential fermentation levels. A clear segregation was observed between fermented and non-fermented tea groups, highlighting pronounced compositional divergences in their metabolite profiles.

In the PCA score plots of positive ions, fresh leaves (FL) and green tea (GT) were observed to be closer to each other compared to oolong tea (OT) and black tea (BT), indicating a higher similarity in their metabolite profiles. This finding was consistent with the results obtained from studies investigating the effects of different tea varieties processed into teas with varying fermentation levels. The comparison of white tea varieties (*Fuyun Six* and *Huangdan*) produced from one bud with two or three leaves was conducted by Li et al. (2020), PCA demonstrated closer positioning of FL to GT relative to BT.

#### 3.3.2 Hierarchical clustering of teas based on fermentation degree

Hierarchical clustering analysis was employed as an additional method to extract information regarding the differences among the four tea groups. The hierarchical clustering analysis results of the positive ion model (Fig. 1B) revealed that FL were clustered closer to GT than to OT and BT, indicating that the metabolite profiles of FL were more similar to those of GT, while a significant divergence was observed between FL and the two fermented teas. This indicated that the hierarchical clustering analysis results were consistent with those of PCA, which effectively differentiated between fermented and non-fermented teas.

#### 3.3.3 Identification of metabolites in different tea types

After the four sets of experimental samples were analyzed using the orthogonal partial least squares discriminant analysis (OPLS-DA) model (Fig. 1C), the cumulative R^2^X value was determined to be 0.807, with the first and second principal components accounting for 58.7% and 13.7% of the total variance, respectively. This explained variance was slightly lower than that obtained from principal component analysis. The OPLS-DA yielded cumulative R^2^Y (goodness of fit) and Q^2^ (predictive goodness of fit) values of 0.682 and 0.741, respectively. Subsequently, the model was internally validated through cross-validation, and a Pcv-ANOVA value of less than 0.05 was obtained, confirming the significance of the model. Additionally, the permutation test results demonstrated that (Fig. 1D), after 200 permutations, R^2^ and Q^2^ values of 0.315 and − 0.663 were achieved, respectively. With R^2^ < 0.4 and Q^2^ < 0.05, it was confirmed that the model was robust and free from overfitting.

VIP values were initially extracted from the variable importance plot of the OPLS-DA model, and pairwise comparisons were conducted between FL, GT, OT, and BT, resulting in six comparison groups. Differential compounds were screened based on VIP > 1 and *p* < 0.05, with the following numbers identified: FL vs GT (223), FL vs OT (253), FL vs BT (292), GT vs OT (313), GT vs BT (296), and OT vs BT (334). These compounds were further analyzed using metabolite databases (mzCloud、MassBank, ChemSpider, and PubChem) Wang et al. (2024), MS/MS spectra, reference standards, and relevant published literature to identify unknown metabolites (J. Gao et al., 2023; Wang et al., 2024;Li et al., 2023).

**Fig. 2:**
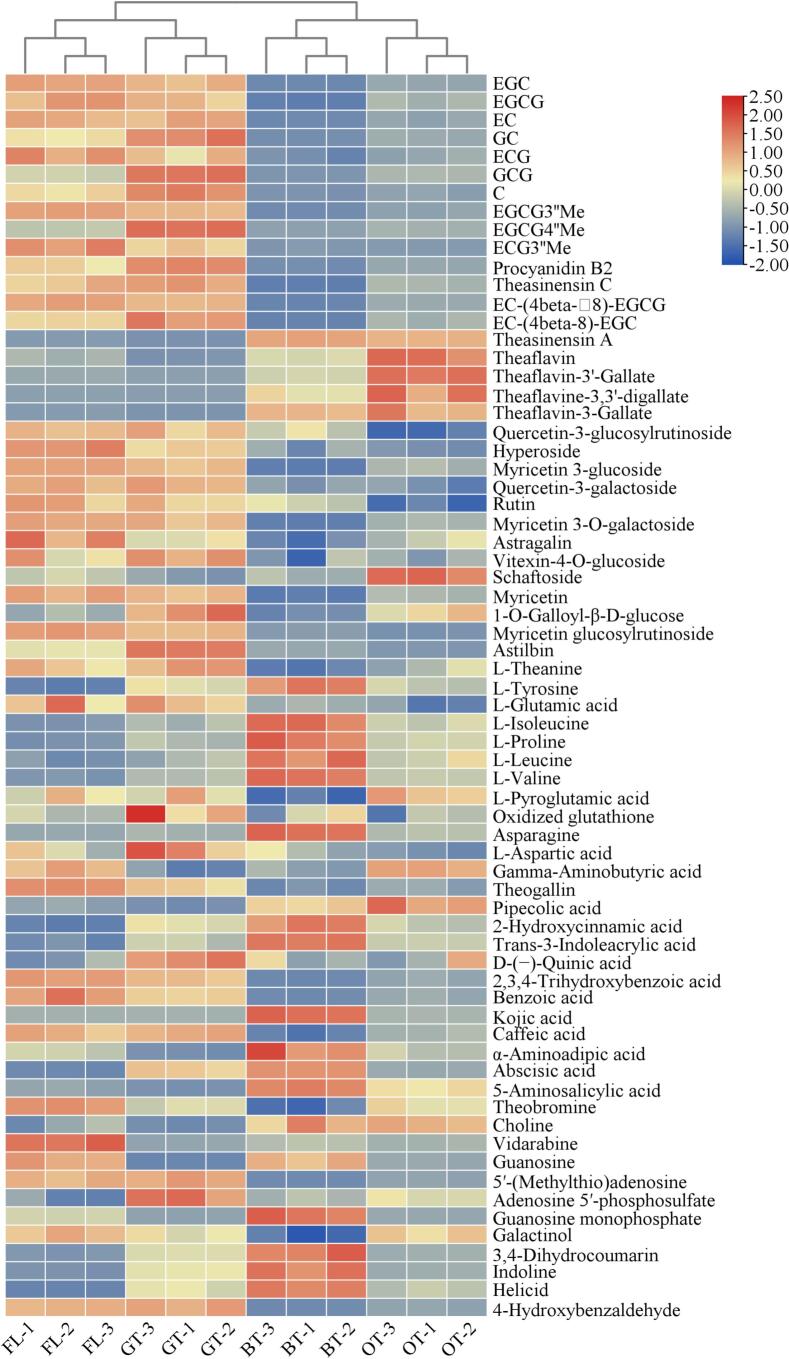
Heat map of differential metabolite content in teas with different degrees of fermentation. Clustering was performed using Euclidean distance. The colour scale represents the *Z*-score normalized intensity of each metabolite (row).

As shown in Table S1, a total of 68 differential compounds, comprising 10 catechins, 9 dimeric catechins, 13 flavonoid glycosides, 12 amino acids, 12 organic acids, 7 alkaloids, and 5 other substances, were screened for structural identification of unknown metabolites based on retention time, exact molecular mass, and characteristic fragment analysis. Heat maps depicting the content of the 68 metabolites were generated, providing a comprehensive overview of the differences among teas with varying degrees of fermentation (Fig. 2).

#### 3.3.4 Differential metabolites among tea types

Catechins, accounted for 60–80% of the total tea polyphenols and were classified as flavan-3-ol compounds. These compounds garnered significant attention in the academic community owing to their notable biological activities, with their pharmacological effects primarily manifested in three areas: significant antioxidant properties(W. Wang et al., 2023), hypoglycemic activity(G. Li, Zhang, Cui, Feng, et al., 2024) and and broad-spectrum antimicrobial effects(Y. Gao et al., 2022). Additionally, catechins were capable of undergoing methylation modifications catalyzed by methyltransferases. This structural modification was found to significantly enhance the biological activity of the compounds. Methylated catechins not only demonstrated improved stability and bioavailability but also exhibited superior physiological functions compared to their non-methylated counterparts, including anti-allergic, anti-obesity, hypoglycemic, hypotensive, and other effects(J. Gao et al., 2023; Kurita et al., 2010). In fresh tea, flavan-3-ols were predominantly present in the 2R, 3R configuration in the absence of heating or other processing(L. Zhang et al., 2019). In non-fermented tea, high levels of flavan-3-ols (2R, 3R) and methylated catechins, such as epigallocatechin-3-(3′′-*O*-methyl) gallate and epigallocatechin-3-(4′′-*O*-methyl) gallate, were retained after fixation and drying. In fermented tea, a significant reduction in catechin and methylated catechin content was observed after rocking, rolling, and fermentation, which was likely associated with the processing methods of oolong tea and black tea. This reduction was attributed to the breakdown of tea leaf cell walls during the rolling stage of fermented tea processing, where catechins were oxidized and polymerized by polyphenol oxidase and peroxidase, leading to the formation of polymers such as TFs, TRs, and TBs, and consequently a significant decrease in catechin content(Zhou et al., 2024). Some studies have also proposed that self-degradation may contribute to this phenomenon(Lv et al., 2006), and further research is required to investigate the effects of withering, fixation, rocking, rolling, and fermentation stages on methylated catechin content.

#### 3.3.5 Metabolomics analysis of dimeric catechins

Nine distinct phenolic compounds were identified across teas with varying fermentation levels, comprising two theasinensins (theasinensin C and A), four TFs (TF, TFDG, TF3’G, TF3G), one proanthocyanidin (procyanidin B2), and two catechin dimers (EC-(4β-8)-EGCG and EC-(4β-8)-EGC). Elevated concentrations of catechin dimers, theasinensin C, and procyanidin B2 were detected in fresh leaves and green tea. Previous studies had demonstrated that anthocyanin concentrations were elevated in fresh leaves and green tea compared to black tea, a finding corroborated by the current experimental data. This observation was potentially attributed to the degradation of anthocyanins and their glycosides during withering and fermentation processes (Li X. et al., 2020; Zhou et al., 2024). Theaflavin and TSA contents were significantly augmented in oolong and black teas relative to non-fermented variants, confirming their status as characteristic phenolic markers of fermented teas. Biosynthetic pathway analysis indicated that these compounds were enzymatically derived from catechin precursors(Aloo et al., 2024). TFs and theasinensins were identified as competitive oxidative derivatives of catechins, with partial oxidation to TFs and reduction to theasinensins occurring during black tea processing, ultimately undergoing coupled oxidation to form TRs (Weerawatanakorn et al., 2015; Xu et al., 2014). Structural characterization revealed that theasinensins were formed through C—C bond-mediated dimerization of catechin B-rings. TSA was characterized as an EGCG dimer containing an *R*-biphenyl linkage, predominantly identified in oolong and black teas(Tanaka et al., 2010; Weerawatanakorn et al., 2015). Comparative analyses had demonstrated that TSA exhibited superior antioxidant efficacy compared to EGCG at equimolar concentrations, attributable to its enhanced hydroxyl group reactivity(W. Wang et al., 2023). Furthermore, TSA was found to possess multimodal glucoregulatory properties through three distinct mechanisms: enhancement of skeletal muscle glucose uptake (Miyata et al., 2013), systemic glucose homeostasis modulation (Qiu et al., 2014), and suppression of α-Glu activity(Tao et al., 2020), and positive correlation with α-Glu suppression as evidenced by Li et al.(G. Li et al., 2025). These findings collectively substantiate the enhanced hypoglycemic potential of fermented tea polyphenols, particularly through TSA mediated metabolic pathways.

#### 3.3.6 Metabolomic analysis of flavonoid glycosides

Flavonoid glycosides, were found to play a dual role in the formation of tea quality. These compounds not only demonstrated significant antioxidant activity but were also identified as key components in determining the flavor profile of tea. Their unique structural characteristics were associated with a low threshold of taste perception, enabling them to produce a pronounced bitter and astringent taste(Guo et al., 2021). In this study, a total of 13 flavonoid glycosides were identified across teas with varying degrees of fermentation. As shown in Fig. 2, the identified flavonoid glycosides included quercetin-3-glucosylrutinoside, hyperoside, myricetin 3-glucoside, quercetin-3-galactoside, rutin, myricetin 3-O-galactoside, astragalin, vitexin-4-O-glucoside, schaftoside, myricetin, 1-O-Galloyl-β-d-glucose, myricetin glucosylrutinoside, and astilbin. The thermogram revealed that flavonoid glycosides were predominantly present in fresh leaves and green tea, with significantly higher content compared to oolong and black tea. It was demonstrated that during tea fermentation, the oxidation of flavonoid glycosides significantly increased due to enzymatic oxidation reactions(Zhou et al., 2024)，and this oxidation process was identified as a key factor contributing to the reduction in flavonoid glycoside content in fermented tea. Fang et al. (Fang et al., 2019) found that the flavonoid glycoside content in fermented teas was significantly lower than that in non-fermented teas through a comparative study of raw materials from the same tea plant species processed into green, oolong, and black teas. An in-depth analysis revealed that the fixation process during green tea processing was a critical control point for maintaining the stability of flavonoid glycosides (Fang et al., 2019). This finding was consistent with the experimental results obtained in this study. However, research gaps remain regarding the chemical properties of flavonoid glycosides and their oxidative transformation mechanisms, particularly the roles of the glycosyl moiety and glycosidic structure in their stability and transformation pathways, which require further in-depth investigation.

#### 3.3.7 Amino acid metabolomics analysis

Amino acids, which were identified as important flavor-presenting substances in tea, were recognized as key components in the chemical composition of tea. The free-state content of amino acids typically accounted for 1% to 4% of the total dry matter of tea leaves. These amino acids not only contributed directly to the fresh flavor characteristics of tea but were also involved in the formation of aroma compounds, which imparted a unique freshness and mellowness to the tea infusion(Zhong et al., 2023; Zhou et al., 2024). From a quality evaluation perspective, amino acid content was positively correlated with tea flavor quality and was commonly regarded as one of the key indicators for assessing tea quality(Horanni & Engelhardt, 2013; Jiang et al., 2019). The experimental results revealed that a variety of amino acids in black tea exhibited a characteristic distribution, with significantly higher levels of L-tyrosine, L-isoleucine, L-proline, L-leucine, L-valine, and asparagine compared to other tea types. Notably, L-theanine, which was identified as a characteristic non-protein amino acid in tea, not only demonstrated significant antioxidant activity but also exhibited neuroprotective effects(M.-Y. Li et al., 2022). Heat map analysis revealed that L-theanine was most abundant in fresh leaves and green tea, while its levels were reduced in oolong and black tea due to the fermentation process. Additionally, γ-aminobutyric acid (GABA), which was recognized as another characteristic non-protein amino acid in tea, played an important role in regulating cardiovascular function, improving sleep quality, and alleviating mental stress(Lin et al., 2012; G. Yang et al., 2023). The heat map analysis indicated that GABA was present at higher levels in fresh leaves and oolong tea, while its content was relatively low in green and black tea.

#### 3.3.8 Metabolomics analysis of organic acids

Phenolic acids, which were classified as aromatic compounds containing carboxyl and hydroxyl functional groups, were found to play a significant role in the formation of tea flavor(Ye et al., 2022). The heat map analysis revealed that the relative contents of theogallin, 2,3,4-trihydroxybenzoic acid, benzoic acid, and caffeic acid were higher in fresh leaves and green tea compared to fermented tea types. In contrast, several phenolic acids exhibited characteristic accumulation in black tea, including pipecolic acid, 2-hydroxycinnamic acid, trans-3-indoleacrylic acid, kojic acid, α-aminoadipic acid, abscisic acid, and 5-aminosalicylic acid, which were present at relatively high levels. This distribution pattern was likely associated with enzymatic oxidation reactions during tea processing.

#### 3.3.9 Alkaloid metabolomics analysis

Caffeine and theobromine, recognized as representative methylxanthine alkaloids, were identified as the predominant purine alkaloids in tea, collectively accounting for approximately 95% of total alkaloid content (Zhou et al., 2024). These methylxanthines were found to serve dual functions as primary bitter agents and key flavor determinants in tea infusion, critically influencing organoleptic quality(Ye et al., 2022). Through metabolomic profiling of differentially fermented tea samples, seven characteristic alkaloids were identified: theobromine, choline, vidarabine, guanosine, 5′-(methylthio)-adenosine, adenosine 5′-phosphosulfate, and guanosine monophosphate. Comparative analyses showed that black tea contained lower levels of theobromine than other tea types. Notably, vidarabine, guanosine and adenosine 5′-phosphosulfate were the most abundant in fresh leaves, whereas the contents of these compounds showed a significant decreasing trend in oolong and black teas after fermentation and processing.

#### 3.3.10 Other categories

A variety of other compounds with characteristic distributions were identified in this study, including galactinol, 3,4-dihydrocoumarin, indoline, helicid, and 4-hydroxybenzaldehyde. The analytical results revealed that 3,4-dihydrocoumarin, indoline, and helicid were present at higher relative levels in black tea compared to other tea types, whereas galactinol exhibited the lowest content in black tea. Notably, 4-hydroxybenzaldehyde was found to be most abundant in non-fermented teas, with its accumulation in fresh leaves and green tea significantly higher than that in fermented teas.

### 3.4 Inhibitory activity of teas on digestive enzymes

The inhibitory activities of α-amylase and α-Glu have long been recognized as potential therapeutic targets for glycaemic management in T2D(G. Li, Zhang, Cui, Feng, et al., 2024). In the present study, we systematically assessed the inhibitory effects of teas with varying degrees of fermentation on both α-amylase and α-Glu. The results of in vitro experiments demonstrated that all tested tea samples exhibited significant dose-dependent inhibitory effects on both enzymes, with α-Glu showing superior inhibitory activity compared to α-amylase.

**Fig. 3:**
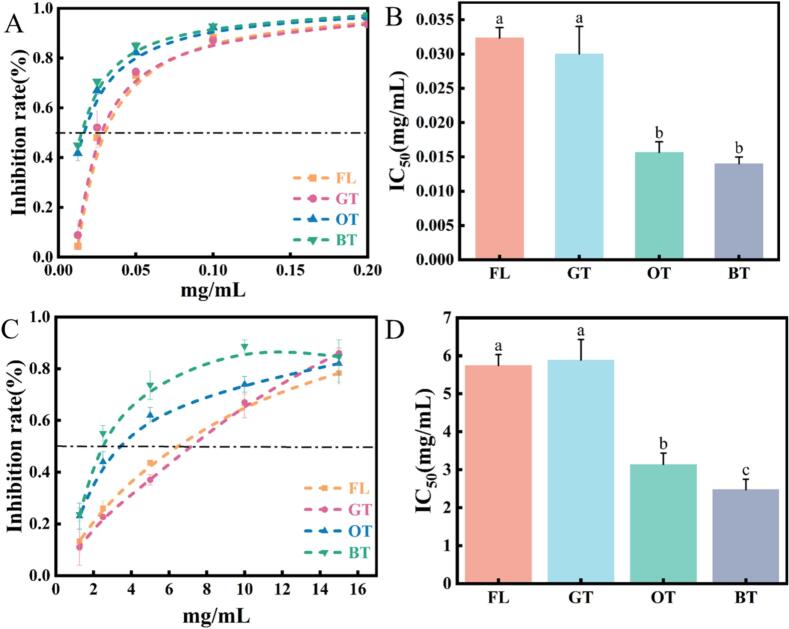
Analysis of inhibition of α-glucosidase(A-B) and α-amylase(C—D) by tea with different fermentation degrees. FL–Fresh Leaves; GT–Green Tea; OT–Oolong Tea; BT–Black Tea. Error bars represent the mean ± standard deviation from three independent experiments. (For interpretation of the references to colour in this figure legend, the reader is referred to the web version of this article.)

As shown in Fig. 3 (A, B), fermented teas were observed to exhibit significantly stronger inhibition of α-Glu compared to non-fermented teas. The IC_50_ values for oolong and black teas were determined to be 0.016 ± 0.0015 mg/mL and 0.014 ± 0.001 mg/mL, respectively, which were significantly lower than those of fresh leaves (0.032 ± 0.0015 mg/mL) and green tea (0.03 ± 0.004 mg/mL). Regarding α-amylase inhibition (Fig. 3 C, D), fermented teas also demonstrated stronger inhibitory activity, with IC_50_ values of 3.14 ± 0.30 mg/mL and 2.48 ± 0.28 mg/mL for oolong and black teas, respectively, which were significantly lower than those of fresh leaves (5.75 ± 0.29 mg/mL) and green tea (5.89 ± 0.54 mg/mL). Notably, black tea was found to exhibit the strongest α-amylase inhibitory activity among all tea samples, with significantly greater activity compared to other tea types (*p* < 0.05). Previous studies have demonstrated that tea extracts exhibit differential inhibitory effects on digestive enzymes. Liu et al. (Liu et al., 2021) confirmed through in vitro experiments that black tea extracts exhibited the most significant inhibitory activity against α-Glu compared to green, oolong, and black tea extracts. This finding was corroborated by a recent study, which demonstrated that black tea extract exhibited the strongest inhibitory effect on α-amylase among six tea types and further revealed a positive correlation between the degree of tea fermentation and α-amylase inhibitory activity(Z. Wang et al., 2024). At the molecular level, studies on the conformational relationships of tea polyphenols have elucidated the chemical basis of their inhibitory effects. In vitro experiments confirmed that TFs, as dimers, exhibited stronger enzyme inhibitory activity compared to catechin monomers. In-depth studies have demonstrated that galloyl groups can precisely target the active site of α-Glu and specifically bind to amino acid residues in the catalytic center through hydrogen bonding and π-π conjugation effects(Sun et al., 2021). These intermolecular interactions were identified as key contributors to the competitive inhibition mechanisms of catechins and TFs.

### 3.5 Correlation between tea compounds and digestive enzyme inhibition

**Fig. 4:**
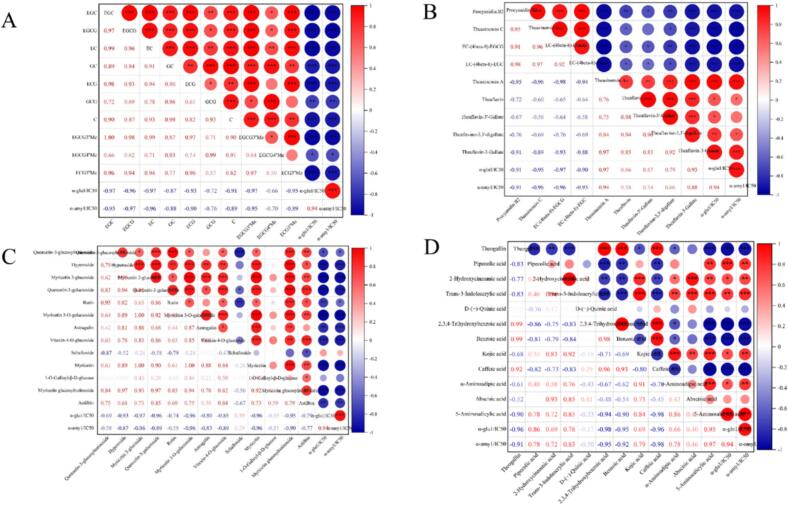
Heatmap of Pearson's correlation analysis of tea catechins (A), dimerised catechins (B), flavonoid glycosides (C), organic acids (D) with digestive enzyme inhibitory activity at different degrees of fermentation. The heatmap displays the pairwise Pearson correlation coefficients (r) based on Z-score normalized abundance data. The colour scale ranges from −1 (strong negative correlation, blue) to +1 (strong positive correlation, red). * *p <* 0.05, ** *p <* 0.01, ** *p < 0.001. (For interpretation of the references to colour in this figure legend, the reader is referred to the web version of this article.)

To investigate the relationship between changes in major differential metabolites and digestive enzyme activities in teas with varying degrees of fermentation, Pearson correlation analyses were conducted. The inhibition rate was expressed as 1/IC_50_, where larger values indicated stronger inhibitory activity (Fig. 4).

Based on the results of Pearson correlation analysis between metabolites and 1/IC_50_ values (Fig. 4), the relationship between different classes of compounds and digestive enzyme inhibitory activity was revealed. The relative contents of the 10 catechin monomers were found to be significantly negatively correlated with digestive enzyme inhibition (correlation coefficients R: −0.66 to −0.97) at significance levels of *p* < 0.01 or *p* < 0.05, indicating that changes in catechin content in tea broths with varying degrees of fermentation significantly influenced digestive enzyme activities (Fig. 4A). Similarly, flavonoid glycosides exhibited trends comparable to those of catechins (Fig. 4D). Among the organic acids, significant differentiation was observed in the correlations between 12 compounds and digestive enzyme inhibitory activity (Fig. 4C). Thea theogallin, quinic acid, 2,3,4-trihydroxybenzoic acid, benzoic acid, and caffeic acid exhibited significant negative correlations with digestive enzyme inhibitory activities (R: −0.91 to −0.96), whereas pipecolic acid, 2-hydroxycinnamic acid, trans-3-Indoleacrylic acid, kojic acid, α-aminoadipic acid, and 5-aminosalicylic acid demonstrated significant positive correlations (R 0.66 to 0.97).

Further analysis revealed that the correlations between the nine dimeric catechins and digestive enzyme inhibitory activity exhibited differentiated patterns. As shown in Fig. 4B, procyanidin B2, theasinensin C, EC-(4β-8)-EGCG, and EC-(4β-8)-EGC exhibited significant negative correlations with digestive enzyme inhibitory activity (R: −0.90 to −0.97). In contrast, TSA, TFs, and their gallate esters (including TF3′G, TFDG, and TF3G) demonstrated significant positive correlations (R: 0.58 to 0.97). Among these compounds, TSA and theaflavin-3-gallate exhibited the most significant correlations with α-Glu inhibitory activity, with correlation coefficients of 0.97 and 0.93 (*p* < 0.001), respectively. Additionally, these two compounds demonstrated highly significant positive correlations with α-amylase inhibitory activity, with correlation coefficients of 0.94 and 0.88 (*p* < 0.001), respectively. The above results indicated that theasinensins and TFs, as characteristic polyphenols of fermented tea, played a key role in digestive enzyme inhibition. This finding explained the stronger digestive enzyme inhibitory activity of fermented tea compared to non-fermented tea at the molecular level. Based on these findings, the effect of TSA gavage on postprandial blood glucose in *db/db* mice was further investigated.

### 3.6 Effect of TSA on blood glucose in *db/db* mice

#### 3.6.1 Effect of TSA on oral starch tolerance in *db/db* mice

The study was conducted using a *db/db* mouse model characterized by genetic defects in the leptin receptor, which exhibited pathological manifestations such as persistent hyperglycemia, hyperlipidemia, and compensated hyperinsulinemia. This model was deemed suitable for evaluating the hypoglycemic activity and metabolic profile of TSA.

**Fig. 5:**
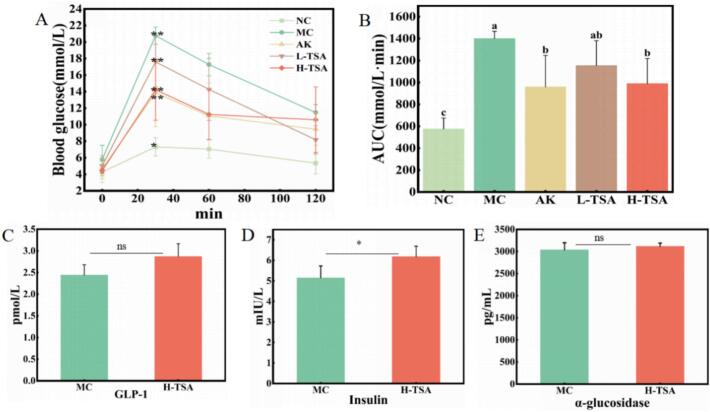
(A) Effects of oral gavage on blood glucose during an oral starch tolerance test in *db/db* mice (*n* = 5); (B) AUC after oral gavage administration of acarbose, L-TSA and H-TSA for 2 h; (C) Effect of TSA on serum GLP-1 in *db/db* mice (*n* = 4); (D) Effect of TSA on serum insulin in *db/db* mice (n = 4); (E) Effect of TSA on alpha glucosidase of intestinal contents in *db/db* mice (n = 4). NC – Normal Control (db/m mice); MC – Model Control (db/db mice); AK – Acarbose treatment group; L-TSA – Low-dose TSA treatment group; H-TSA – High-dose TSA treatment group. 5 A represents blood glucose levels at different time points (0 min vs. 30 min), while Fig. 5C, D, and E represent comparisons between experimental(H-TSA) and control groups(MC) at the 30-min time point. The error bars in Figs. 5A/B represent the mean ± standard deviation from five independent experiments; the error bars in Figs. 5C/D/E represent the mean ± standard deviation from four independent experiments.

The results of the oral glucose tolerance test (Fig. 5A) revealed that blood glucose levels in mice after starch loading followed a typical dynamic trajectory, peaking at 30 min and then gradually declining, which reflected the body's glucose metabolism regulation capacity. Quantitative analysis indicated that, compared to the group (MC, AUC = 1400.63 ± 66.32 mmol/L·min), the L-TSA group (AUC = 1154.25 ± 227.32 mmol/L·min) exhibited a trend toward reduced blood glucose levels, although the difference was not statistically significant (*p >* 0.05, Fig. 5B). Notably, the AUC value in the positive control acarbose group was significantly reduced to 959.63 ± 286.85 mmol/L·min (*p* < 0.05). Further analysis demonstrated that the high-dose TSA intervention group (AUC = 990 ± 228.67 mmol/L·min) exhibited a significant glucose-lowering effect (*p* < 0.05), with an efficacy comparable to that of acarbose. The above results confirmed that TSA intervention dose-dependently improved postprandial glucose homeostasis in *db/db* mice.

#### 3.6.2 Effect of TSA on alpha glucosidase of intestinal contents in *db/db* mice

This study further examined the α-Glu secretion levels in the intestinal contents 30 min after a meal to elucidate the glucose-lowering mechanism of TSA. The results demonstrated (Fig. 5E) that α-Glu secretion levels were 3035 ± 162.7 pg/mL in the model control group and 3118.3 ± 72.5 pg/mL in the high-dose TSA group. There was no statistically significant difference between the two groups (*p >* 0.05). This finding suggests that α-Glu secretion levels are not the primary mechanism underlying the hypoglycaemic effect of TSA.

#### 3.6.3 Effect of TSA on serum GLP-1 in *db/db* mice

GLP-1, an important enteric proinsulin hormone secreted by ileal cells, was recognized for its role in regulating feeding behavior through delayed gastric emptying and increased satiety. To investigate the hypoglycemic mechanism of TSA, serum GLP-1 levels were measured at peak blood glucose (30 min postprandial) in this study (Fig. 5C). The results demonstrated that the GLP-1 concentration in the high-dose TSA group (2.87 ± 0.29 pmol/L) was elevated compared to the model control group (2.44 ± 0.24 pmol/L), although the difference was not statistically significant (*p >* 0.05). This finding suggested that the glucose-lowering effect of TSA at 30 min postprandial might be independent of the GLP-1 secretory pathway, and further investigation is required to elucidate its specific mechanism of action.

#### 3.6.4 Effect of TSA on serum insulin in *db/db* mice

As a central regulator of pancreatic β-cell secretion, insulin was recognized to serve a critical function in glucose homeostasis maintenance. Pathological manifestations of insulin dysfunction were characterized by two principal mechanisms: impairments in insulin secretion and reduced biological efficacy of insulin (termed insulin resistance)(G. Li, Zhang, Cui, Feng, et al., 2024). Both pathological manifestations were demonstrated to disrupt glucose homeostasis, ultimately resulting in hyperglycemic states. In the present investigation (Fig. 5D), elevated serum insulin levels were observed following high-dose TSA intervention (6.18 ± 0.51 mIU/L), with statistical significance (*p* < 0.05) when compared to the model control group (5.15 ± 0.58 mIU/L). These findings indicated that the hypoglycemic effect of TSA at 30 min postprandial was primarily mediated through enhanced insulin secretion. Previous experimental evidence had established that the antidiabetic activity of black tea extract was predominantly associated with stimulation of insulin secretion, an effect that was previously correlated with the high theaflavin content of the extract(Tang et al., 2013). The current experimental data further suggested that, in addition to TFs, this biological activity might be attributable to TSA components.

#### 3.6.5 Effect of TSA on serum metabolic profiles in *db/db* mice

##### 3.6.5.1 Serum metabolic profile

**Fig. 6:**
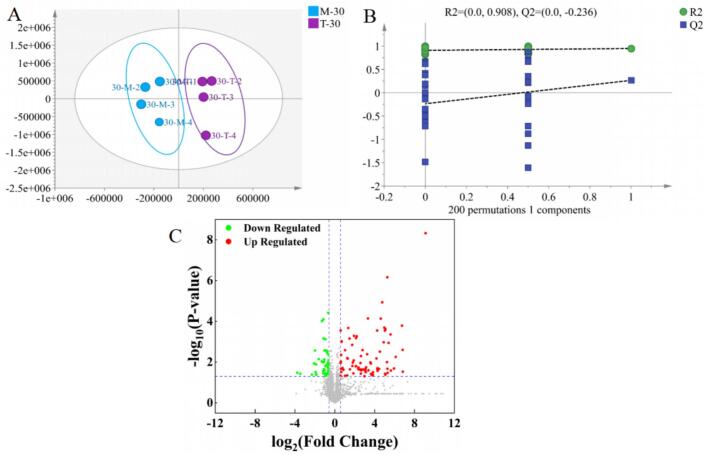
(A) OPLS-DA plot comparing the H-TSA group and the control group in *db/db* mice(n = 4); (B)200 times validation plot analysis;(C) Volcano plots of control group and H-TSA group serum metabolome difference analysis, Log₂(Fold Change: H-TSA / Control). The fold change (FC) is calculated as H-TSA / MC. FC > 1.5 and *p* < 0.05 indicates that the metabolite is up-accumulated in the H-TSA group relative to the MC group(red). FC < 0.67and *p* < 0.05 indicates that the metabolite is down-accumulated in the H-TSA group(green) (up-accumulated in the MC group). (For interpretation of the references to colour in this figure legend, the reader is referred to the web version of this article.)

Based on the above findings, high-dose TSA was found to significantly reduce AUC values by promoting insulin secretion and enhancing glucose uptake and utilization in peripheral tissues during the postprandial glucose peak (30 min). To further elucidate the glucose-lowering mechanism of TSA, a systematic metabolomics analysis of serum small molecule metabolites was conducted at 30 min postprandial in the MC group and the H-TSA group. OPLS-DA was employed to assess the differences in serum metabolic profiles between the two groups (Fig. 6A, B). The OPLS-DA score plots revealed significant spatial separation between the MC group and the H-TSA group (Fig. 6A), indicating that the TSA intervention significantly altered the serum metabolic profile of the mice. To validate the reliability of the model, a 200-permutation test was conducted (Fig. 6B). The results demonstrated that Q^2^ < 0 and that the values of R^2^ and Q^2^ decreased with reduced replacement retention, confirming that the OPLS-DA model was not overfitted and exhibited high reliability(Luo et al., 2023). To further screen for differential metabolites, volcano plots were constructed for visualization and analysis (Fig. 6C). The screening criteria were defined as significantly up-regulated metabolites (FC > 1.5, *p* < 0.05) and significantly down-regulated metabolites (FC < 0.67, *p* < 0.05), which were labeled in red and green, respectively.

##### 3.6.5.2 Identification of differential serum metabolites

As presented in Table S2, the differential metabolites were categorized into four principal classes: (a) phospholipids, exemplified by PC (36:5), LysoPC (0:0/18:1(9Z)), and SM (d18:0/16:1(9Z)); (b) lysophospholipids, including LysoPC(15:0/0:0) and LysoPC(14:0/0:0); (c) fatty acids and derivatives, represented by unsaturated fatty acids and acylcarnitines; and (d) amino acids and derivatives, such as 3-Aminocaproic acid and Indole-3-carboxaldehyde.

It was established through previous investigations that lysophospholipids were critically involved in the modulation of inflammatory responses(D'Arrigo & Servi, 2010; Frasch & Bratton, 2012) and obesity pathogenesis (Bellot et al., 2023; Rubio-Rodríguez et al., 2021). The biological significance of unsaturated fatty acids and their derivatives was recognized as essential components of lipid metabolism, with specific derivatives (e.g., prostaglandins) being identified as key mediators in inflammatory and immune regulatory processes(Dyall et al., 2022). With regard to amino acid metabolism, a substantial association was reported between microbiota-related amino acid metabolites and T2D, wherein metabolic dysregulation of amino acids was documented to precede T2D onset(Karusheva et al., 2019; S. J. Yang et al., 2018). In an experimental study examining functional oligosaccharides, stachyose administration was shown to enhance indole derivative production, particularly indole-3-carboxaldehyde, while concurrently improving intestinal function and glucose metabolism(Yan et al., 2023). Comparative analysis between the MC and H-TSA groups revealed that serum differential compounds were predominantly associated with three metabolic pathways: lipid metabolism (encompassing lysophosphatidylinositol, phospholipids, sphingolipids, and acylcarnitines), amino acid metabolism, and fatty acid metabolism. These observations suggested that TSA intervention dynamically reprograms key metabolic pathways, particularly those involving lipid and amino acid metabolism.

#### 3.6.6 Analysis of TSA metabolites in *db/db* mice serum

**Fig. 7:**
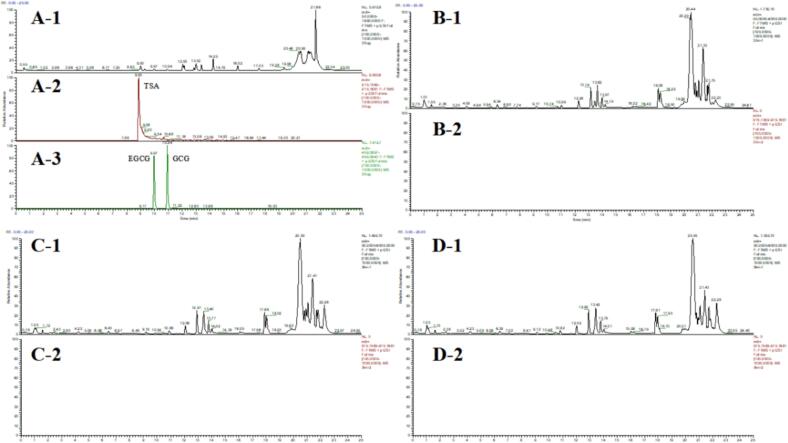
(A) Characterization of standard compounds. (A-1) Total ion chromatogram (TIC) of a mixed standard solution (0–25 min). (A-2/3) Chromatographic peaks and retention times of individual standards: TSA (8.85 min), EGCG (9.97 min), and GCG (10.94 min). (B—D) Analysis of serum samples from db/db mice after administration. (B-1, C-1, D-1) TIC of serum at 30, 180, and 300 min, respectively. (B-2, C-2, D-2) Extracted ion chromatograms (XICs) for TSA (*m*/*z* 915.1635, ±5 ppm) in serum at the corresponding time points. The absence of a detectable TSA peak, in contrast to the standard in (A-2), indicates that TSA was not absorbed into systemic circulation.

TSA metabolism was investigated in *db/db* mice at 30, 180, and 300 min post-gavage, and further analyzed in the serum of *db/db* mice (Fig. 7). The serum metabolic profiles of mice were analyzed at 30, 180, and 300 min post-oral administration. Components with *m*/*z* 915.1635 (TSA, ± 5 ppm) were extracted in positive ion mode, but the monomeric form of TSA was not detected. Similarly, components with m/z 459.0920 (EGCG, ±5 ppm) were extracted and analyzed, but no detection was observed. In contrast, in an in vivo experiment using Sprague-Dawley rats, plasma components were analyzed using electrochemical detection-high performance liquid chromatography (ECD-HPLC) following oral administration of theasinensins, demonstrating that TSB and TSA were absorbed into the bloodstream in their intact forms(Qiu et al., 2012). This inconsistency was hypothesized to result from the following factors: (1) interspecies differences in in vivo metabolism may be attributed to the significantly reduced intestinal absorption capacity in *db/db* model mice compared to healthy Sprague-Dawley rats, which resulted in undetectable TSA levels; (2) variations in the analyzed substrates may also account for the observed discrepancies, as TSA content in this study was measured in the serum of *db/db* mice, whereas Qiu et al. quantified TSA in the plasma of Sprague-Dawley rats; (3) the low intestinal bioavailability of TSA, an active molecule derived from fermented tea, further limited its absorption and utilization. This is likely due to its relatively high molecular weight, in contrast to smaller molecules such as EGCG and EGC, which are more readily absorbed in the intestine(Qiu et al., 2012).

## 4 Conclusion

This study systematically clarifies the effects of different processing techniques on the digestive enzyme inhibitory activity of tea and its key active components. Metabolomics analysis revealed that the characteristic component TSA in fermented teas (oolong tea and black tea) is one of the key active molecules most strongly associated with in vitro digestive enzyme inhibitory activity. In vivo experiments confirmed that TSA effectively improves postprandial blood glucose in *db/db* mice. Its mechanism primarily relies on promoting insulin secretion rather than directly inhibiting intestinal α-glucosidase activity. Serum metabolomics further indicated that TSA intervention significantly modulates amino acid metabolism and fatty acid metabolism pathways, which are closely related to glycolipid metabolism. This provides direct scientific evidence for utilizing tea beverages, particularly fermented teas, as a dietary adjunct for blood glucose management in individuals with prediabetes and T2D. It also points the way for developing functional tea products using TSA as a marker. Given the limitation of oral bioavailability of TSA, future studies with an adequate animal sample size could explore novel delivery systems (e.g., encapsulation technologies) to enhance its efficacy. Furthermore, in-depth evaluation of its distribution across various tissues and organs, along with its long-term safety, is warranted to facilitate its translation into clinical applications.

## Statement

During the preparation of this work the authors used Deepseek in order to improve language. After using this tool the authors reviewed and edited the content as needed and take full responsibility for the content of the published article.

## CRediT authorship contribution statement

**Guangneng Li:** Writing – original draft, Validation, Investigation, Formal analysis, Data curation. **Jianyong Zhang:** Writing – original draft, Validation, Investigation, Formal analysis, Data curation. **Ying Gao:** Validation, Methodology, Investigation. **Hongchun Cui:** Validation, Investigation. **Debao Niu:** Writing – review & editing, Supervision, Resources. **Junfeng Yin:** Writing – review & editing, Project administration, Funding acquisition, Conceptualization.

## Ethics statement

All animal experiments were performed in compliance with the relevant laws and institutional guidelines for the care and use of laboratory animals in China (GB/T 39760–2021) and were approved by the Laboratory Animal Management and Ethics Committee of Zhejiang Chinese Medical University (Animal Ethics Approval Number: SYXK (ZHE) 2021–0012).

## Declaration of competing interest

The authors declare that they have no known competing financial interests or personal relationships that could have appeared to influence the work reported in this paper.

## Data Availability

Data will be made available on request.

## Appendix A Supplementary data

Supplementary material Image 1
