# Supplementary material for: From chemical characterization to functional validation: Screening of anti-hyperglycemic components and their action mechanisms in differentially fermented teas
Source: Food Chem X. 2026 Mar 6;35:103721. doi: 10.1016/j.fochx.2026.103721 (PMC12994079; doi:10.1016/j.fochx.2026.103721)

# Table S1

**Table S1 Identification of differential compounds between teas with different degrees of fermentation**

| Primary ID | Formula | M+H | RT(min) | CAS | Name |
| --- | --- | --- | --- | --- | --- |
|  |  |  | **Catechins** |  |  |
| 4 | C_15_H_15_O_7_ | 307.0807 | 7.226 | [970-74-1](https://commonchemistry.cas.org/detail?cas_rn=970-74-1&search=970-74-1) | EGC |
| 5 | C_22_H_19_O_11_ | 459.09172 | 10.061 | [989-51-5](https://commonchemistry.cas.org/detail?cas_rn=989-51-5&search=989-51-5) | EGCG |
| 10 | C_15_H_15_O_6_ | 291.08586 | 9.836 | [490-46-0](https://commonchemistry.cas.org/detail?cas_rn=490-46-0&search=490-46-0) | EC |
| 13 | C_15_H_15_O_7_ | 307.08073 | 4.626 | [970-73-0](https://commonchemistry.cas.org/detail?cas_rn=970-73-0&search=970-73-0) | GC |
| 15 | C_22_H_19_O_10_ | 443.09672 | 12.931 | [1257-08-5](https://commonchemistry.cas.org/detail?cas_rn=1257-08-5&search=1257-08-5) | ECG |
| 20 | C_22_H_19_O_11_ | 459.09172 | 11.039 | [4233-96-9](https://commonchemistry.cas.org/detail?cas_rn=4233-96-9&search=4233-96-9) | GCG |
| 54 | C_15_H_15_O_6_ | 291.08588 | 7.677 | [154-23-4](https://commonchemistry.cas.org/detail?cas_rn=154-23-4&search=154-23-4) | C |
| 114 | C_23_H_21_O_11_ | 473.10757 | 12.332 | [83104-87-4](https://commonchemistry.cas.org/detail?cas_rn=83104-87-4) | EGCG3''Me |
| 356 | C_23_H_21_O_11_ | 473.10756 | 13.459 | [224434-07-5](https://commonchemistry.cas.org/detail?cas_rn=224434-07-5) | EGCG4''Me |
| 454 | C_23_H_21_O_10_ | 457.11249 | 14.291 | [83104-86-3](https://commonchemistry.cas.org/detail?cas_rn=83104-86-3&search=Epicatechin%203-O-(3-O-methyl)gallate) | ECG3''Me |
| **Dimeric catechins** | | | | | |
| 31 | C_30_H_27_O_12_ | 579.14924 | 8.689 | 29106-49-8 | Procyanidin B2 |
| 39 | C_30_H_27_O_14_ | 611.13902 | 5.523 | 89013-69-4 | Theasinensin C |
| 101 | C_37_H_31_O_17_ | 747.15452 | 9.844 | / | EC-(4beta-8)-EGCG |
| 106 | C_30_H_27_O_13_ | 595.14429 | 7.32 | / | EC-(4beta-8)-EGC |
| 184 | C_44_H_35_O_22_ | 915.16055 | 8.978 | 89064-31-3 | Theasinensin A |
| 286 | C_29_H_25_O_12_ | 565.1339 | 16.271 | [4670-05-7](https://commonchemistry.cas.org/detail?cas_rn=4670-05-7&search=4670-05-7) | Theaflavin |
| 471 | C_36_H_29_O_16_ | 717.14437 | 16.885 | [28543-07-9](https://pubchem.ncbi.nlm.nih.gov/substance/254772951) | Theaflavin-3'-gallate |
| 583 | C_43_H_33_O_20_ | 869.15519 | 16.883 | [33377-72-9](https://commonchemistry.cas.org/detail?cas_rn=33377-72-9&search=33377-72-9) | Theaflavine-3,3'-digallate |
| 600 | C_36_H_29_O_16_ | 717.14441 | 16.648 | [30462-34-1](https://commonchemistry.cas.org/detail?cas_rn=30462-34-1&search=30462-34-1) | Theaflavin-3-gallate |
| **Flavonoid glycosides** | | | | | |
| 24 | C_33_H_41_O_21_ | 773.21251 | 12.777 | / | Quercetin-3-glucosylrutinoside |
| 51 | C_21_H_21_O_12_ | 465.10236 | 13.403 | 482-36-0 | Hyperoside |
| 76 | C_21_H_21_O_13_ | 481.09736 | 12.102 | 19833-12-6 | Myricetin 3-glucoside |
| 79 | C_21_H_21_O_12_ | 465.10236 | 13.217 | 482-36-0 | Quercetin-3-galactoside |
| 96 | C_27_H_31_O_16_ | 611.16015 | 13.157 | 153-18-4 | Rutin |
| 113 | C_21_H_21_O_13_ | 481.09736 | 11.903 | 15648-86-9 | Myricetin 3-O-galactoside |
| 120 | C_21_H_21_O_11_ | 449.10747 | 14.281 | 480-10-4 | Astragalin |
| 139 | C_27_H_31_O_15_ | 595.16521 | 12.77 | 76135-82-5 | Vitexin-4-O-glucoside |
| 142 | C_26_H_29_O_14_ | 565.15474 | 12.119 | 51938-32-0 | Schaftoside |
| 317 | C_15_H_11_O_8_ | 319.04426 | 12.102 | 529-44-2 | Myricetin |
| 344 | C_13_H_17_O_10_ | 333.08119 | 1.969 | 13405-60-2 | 1-O-Galloyl-β-D-glucose |
| 656 | C_33_H_41_O_22_ | 789.20754 | 11.516 | / | Myricetin glucosylrutinoside |
| 818 | C_21_H_23_O_11_ | 451.1233 | 5.769 | 29838-67-3 | Astilbin |
| **Amino acids** | | | | | |
| 2 | C_7_H_15_ N_2_O_3_ | 175.10745 | 1.023 | 3081-61-6 | L-Theanine |
| 60 | C_9_H_12_ NO_3_ | 182.08096 | 1.581 | 60-18-4 | L-Tyrosine |
| 67 | C_5_H_10_ NO_4_ | 148.06018 | 0.687 | 56-86-0 | L-Glutamic acid |
| 68 | C_6_H_14_ NO_2_ | 132.10175 | 1.586 | 73-32-5 | L-Isoleucine |
| 69 | C_5_H_10_ NO_2_ | 116.07057 | 0.741 | 147-85-3 | L-Proline |
| 73 | C_6_H_14_ NO_2_ | 132.10175 | 1.728 | [61-90-5](https://commonchemistry.cas.org/detail?cas_rn=61-90-5&search=61-90-5) | L-Leucine |
| 75 | C_5_H_2_ NO_2_ | 118.08623 | 0.931 | [72-18-4](https://commonchemistry.cas.org/results?q=L-Valine) | L-Valine |
| 135 | C_5_H_8_ NO_3_ | 130.0497 | 0.679 | [98-79-3](https://commonchemistry.cas.org/detail?cas_rn=98-79-3&search=98-79-3) | L-Pyroglutamic acid |
| 176 | C_20_H_33_N_6_O_12_S_2_ | 613.1572 | 1.537 | [27025-41-8](https://commonchemistry.cas.org/detail?cas_rn=27025-41-8&search=27025-41-8) | Oxidized glutathione |
| 232 | C_4_H_9_N_2_O_3_ | 133.06061 | 0.674 | [70-47-3](https://commonchemistry.cas.org/detail?cas_rn=70-47-3&search=70-47-3) | Asparagine |
| 419 | C_4_H_8_NO_4_ | 134.04463 | 0.676 | [56-84-8](https://commonchemistry.cas.org/detail?cas_rn=56-84-8&search=56-84-8) | L-Aspartic acid |
| 476 | C_4_H_10_NO_2_ | 104.07072 | 0.691 | [56-12-2](https://commonchemistry.cas.org/detail?cas_rn=56-12-2&search=Gamma-Aminobutyric%20acid) | Gamma-Aminobutyric acid |
| **Organic acids** | | | | | |
| 8 | C_14_H_17_O_10_ | 345.08103 | 2.975 | 17365-11-6 | Theogallin |
| 17 | C_6_H_12_NO_2_ | 130.08612 | 2.686 | [1723-00-8](https://commonchemistry.cas.org/detail?cas_rn=1723-00-8&search=1723-00-8) | Pipecolic acid |
| 56 | C_9_H_9_O_3_ | 165.05438 | 1.575 | [614-60-8](https://commonchemistry.cas.org/detail?cas_rn=614-60-8&search=614-60-8) | 2-Hydroxycinnamic acid |
| 66 | C_11_H_10_NO_2_ | 188.07037 | 5.607 | [29953-71-7, 1204-06-4](https://commonchemistry.cas.org/detail?cas_rn=29953-71-7&search=29953-71-7) | Trans-3-Indoleacrylic acid |
| 81 | C_7_H_13_O_6_ | 193.07036 | 0.767 | [77-95-2](https://commonchemistry.cas.org/detail?cas_rn=77-95-2&search=77-95-2) | Quinic acid |
| 82 | C_7_H_7_O_5_ | 171.02852 | 2.975 | [610-02-6](https://commonchemistry.cas.org/detail?cas_rn=610-02-6&search=610-02-6) | 2,3,4-Trihydroxybenzoic acid |
| 91 | C_7_H_7_O_2_ | 123.04392 | 12.93 | [65-85-0](https://commonchemistry.cas.org/detail?cas_rn=65-85-0&search=65-85-0) | Benzoic acid |
| 188 | C_6_H_7_O_4_ | 143.0337 | 4.612 | [501-30-4](https://commonchemistry.cas.org/detail?cas_rn=501-30-4&search=501-30-4) | Kojic acid |
| 234 | C_9_H_9_O_4_ | 181.0489 | 7.222 | [331-39-5](https://commonchemistry.cas.org/detail?cas_rn=331-39-5&search=331-39-5) | Caffeic acid |
| 306 | C_6_H_12_NO_4_ | 162.07584 | 0.747 | [1118-90-7](https://commonchemistry.cas.org/detail?cas_rn=1118-90-7&search=1118-90-7) | α-Aminoadipic acid |
| 474 | C_15_H_21_O_4_ | 265.14297 | 16.051 | [21293-29-8](https://commonchemistry.cas.org/detail?cas_rn=21293-29-8&search=Abscisic%20acid) | Abscisic acid |
| 617 | C_7_H_8_NO_3_ | 154.04959 | 1.178 | [89-57-6](https://commonchemistry.cas.org/detail?cas_rn=89-57-6&search=89-57-6) | 5-Aminosalicylic acid |
| **Alkaloids** | | | | | |
| 16 | C_7_H_9_N_4_O_2_ | 181.07173 | 4.783 | [83-67-0](https://commonchemistry.cas.org/detail?cas_rn=83-67-0&search=83-67-0) | Theobromine |
| 27 | C_5_H_14_NO | 104.107 | 0.676 | [62-49-7](https://commonchemistry.cas.org/detail?cas_rn=62-49-7&search=62-49-7) | Choline |
| 29 | C_10_H_14_N_5_O_4_ | 268.10359 | 2.087 | 5536-17-4 | Vidarabine |
| 140 | C_10_H_14_N_5_O_5_ | 284.09852 | 2.354 | 118-00-3 | Guanosine |
| 195 | C_11_H_16_N_5_O_3_S | 298.09649 | 6.229 | [2457-80-9](https://commonchemistry.cas.org/detail?cas_rn=2457-80-9&search=2457-80-9) | 5′-(Methylthio)adenosine |
| 302 | C_10_H_15_N_5_O_10_PS | 348.0698 | 1.017 | 485-84-7 | Adenosine 5′-phosphosulfate |
| 357 | C_10_H_15_N_5_O_8_P | 364.06487 | 1.342 | 85-32-5 | Guanosine monophosphate |
| **Others** | | | | | |
| 11 | C_12_H_23_O_11_ | 343.12294 | 0.774 | [3687-64-7](https://commonchemistry.cas.org/detail?cas_rn=3687-64-7&search=3687-64-7) | Galactinol |
| 47 | C_9_H_9_O_2_ | 149.05949 | 3.272 | 119-84-6 | 3,4-Dihydrocoumarin |
| 59 | C_8_H_10_N | 120.08071 | 3.277 | 496-15-1 | Indoline |
| 351 | C_13_H_17_O_7_ | 123.04397 | 1.571 | 80154-34-3 | Helicid |
| 517 | C_7_H_7_O_2_ | 123.04391 | 9.843 | [123-08-0](https://commonchemistry.cas.org/detail?cas_rn=123-08-0&search=123-08-0) | 4-Hydroxybenzaldehyde |

# Table S2

**Table S2 Differential metabolites in MC and H-TSA groups**

| Primary ID | Compound | [M+H] | RT (min) | Fragment | HMDB ID | *p* | FC | Formula | Classification |  |  |
| --- | --- | --- | --- | --- | --- | --- | --- | --- | --- | --- | --- |
| **Down** | | | | | | | | | |  |  |
| 109 | C_6_H_14_NO_2_ | 132.10171 | 2.58 | 86.0969;69.0704;132.1020 | HMDB0029168 | 0.04 | 0.27 | ^△^3-Aminocaproic acid | Amino Acid Derivatives |  |  |
| 202 | C_23_H_45_NO_7_P | 478.29234 | 20.803 | 337.2737;95.0859;81.0703；263.2369 | HMDB0011507 | 0.02 | 0.63 | ^△^LysoPE(18:2(9Z,12Z)/0:0) | Lysophospholipids |  |  |
| 242 | C_24_H_39_O_4_ | 391.28384 | 21.088 | 149.0235;71.0680；391.2843 | HMDB0000503 | 0.04 | 0.63 | ^△^7alpha-Hydroxy-3-oxo-5beta-cholan-24-oic acid | Bile Acid Derivatives |  |  |
| 453 | C_26_H_53_NO_7_P | 522.35536 | 21.25 | 184.0734;104.1073;86.0968 | HMDB0061701 | 0.00 | 0.52 | ^△^LysoPC(0:0/18:1(9Z)) | Lysophospholipids |  |  |
| 660 | C_18_H_33_O_3_ | 297.24104 | 21.424 | 261.2213;297.2424;219.2111 | HMDB0340917 | 0.03 | 0.45 | ^△^(11E,13Z)-10-hydroxyoctadeca-11,13-dienoic acid | Unsaturated Fatty Acids |  |  |
| 718 | C_23_H_49_NO_7_P | 482.32388 | 20.66 | 184.0735;104.1074;86.0969；299.2582；464.3139 | HMDB0010381 | 0.01 | 0.65 | ^△^LysoPC(15:0/0:0) | Lysophospholipids |  |  |
| 927 | C_23_H_44_NO_4_ | 398.32598 | 20.54 | 85.0284，398.3265；3392535； | HMDB0013207 | 0.04 | 0.50 | ^△^9-Hexadecenoylcarnitine | Acylcarnitines |  |  |
| 984 | C_48_H_83_NO_8_P | 832.5874 | 12.432 | 832.5889;614.1927;519.1710;363.8385 | HMDB0289823 | 0.02 | 0.65 | ^△^PC(P-18:1(9Z)/22:5(4Z,7Z,10Z,13Z,19Z)-O(16,17)) | Phospholipids |  |  |
| 1423 | C_22_H_47_NO_7_P | 468.30819 | 20.12 | 86.0964；104.1070；146.9818；285.2424；307.2244； | HMDB0010379 | 0.01 | 0.27 | #^△^LysoPC(14:0/0:0) | Lysophospholipids |  |  |
| 1657 | C_24_H_37_O_3_ | 373.27333 | 16.907 | 355.2632;161.1327;107.0858 | HMDB0013627 | 0.00 | 0.63 | ^△^Cervonoyl ethanolamide | Ethanolamides |  |  |
| **Up** | | | | | | | | | | |  |
| 11 | C_26_H_55_NO_7_P | 524.37072 | 19.803 | 184.0734;104.1073;86.0968；240.0985；258.1095；341.3048；506.3610，524.3610 | HMDB0258493 | 0.02 | 4.29 | ^△^2-Lysophosphatidylcholine | Lysophospholipids |  |  |
| 115 | C_44_H_79_NO_8_P | 780.5508 | 22.055 | 184.0736；125.0000；86.0969；  597.4753 | HMDB0007890 | 0.02 | 61.32 | ^△^PC(36:5) | Phospholipids |  |  |
| 121 | C_46_H_81_NO_8_P | 806.5677 | 22.052 | 184.0736；125.0000；86.0969；250.0357；351.6247；424.4361 | HMDB0007991 | 0.01 | 69.38 | ^△^PC(38:6) | Phospholipids |  |  |
| 150 | C_27_H_51_N_5_O_3_P | 524.371 | 22.476 | 184.0734;  104.1073；  86.0968 | HMDB0011128 | 0.04 | 14.17 | ^△^LysoPC(0:0/18:0) | Lysophospholipids |  |  |
| 154 | C_44_H_81_NO_8_P | 782.56814 | 22.082 | 184.0736；125.0000；86.0969； | HMDB0007889 | 0.02 | 38.95 | ^△^PC(36:4) | Phospholipids |  |  |
| 294 | C_48_H_83_NO_8_P | 832.5841 | 21.28 | 184.0736；125.0000；86.0969； | HMDB0289823 | 0.03 | 10.37 | ^△^PC(P-18:1(9Z)/22:5(4Z,7Z,10Z,13Z,19Z)-O(16,17)) | Phospholipids |  |  |
| 320 | C_39_H_80_N_2_O_6_P | 703.57424 | 22.171 | 184.0736；125.0000；86.0969； | HMDB0013464 | 0.02 | 3.39 | ^△^SM(d18:0/16:1(9Z)) | Sphingolipids |  |  |
| 424 | C_42_H_79_NO_8_P | 756.55307 | 22.151 | 184.0736；125.0000；86.0969； | HMDB0007881 | 0.00 | 3.80 | ^△^PC(34:3) | Phospholipids |  |  |
| 466 | C_29_H_58_O_8_P | 565.38649 | 21.989 | 284.2948；373.2115，393.2617；127.1118；421.2914 | HMDB0115489 | 0.01 | 11.92 | ^△^PA(8:0/18:0) | Phospholipids |  |  |
| 468 | C_24_H_53_NO_6_P | 482.36027 | 21.568 | 341.3050；310.3105；104.1073；184.0735； | HMDB0243890 | 0.00 | 4.40 | ^△^1-O-Hexadecyl-sn-glycero-3-phosphocholine | Phospholipids |  |  |
| 478 | C_9_H_8_NO | 146.05972 | 6.266 | 128.9510；97.0080；91.0547 | HMDB0029737 | 0.00 | 552.05 | ^△^Indole-3-carboxaldehyde | Indole Derivatives |  |  |
| 536 | C_25_H_53_NO_7_P | 510.35525 | 21.456 | 184.0735；104.1074；256.2636 | HMDB0012108 | 0.05 | 2.39 | ^△^LysoPC(17:0/0:0) | Lysophospholipids |  |  |
| 654 | C_48_H_79_NO_8_P | 828.55008 | 21.265 | 146.9819，86.0969；  645.4855 | HMDB0113688 | 0.00 | 32.71 | ^△^PE-NMe(22:6(4Z,7Z,10Z,13Z,16Z,19Z)/20:3(5Z,8Z,11Z)) | Phospholipids |  |  |
| 781 | C_26_H_55_NO_6_P | 508.37599 | 22.208 | 184.0735；104.1074；86.0969； | HMDB0013122 | 0.02 | 1.83 | ^△^LysoPC(P-18:0/0:0) | Lysophospholipids |  |  |
| 859 | C_33_H_57_O_6_ | 549.4123 | 21.88 | 104.1074；184.0735；355.2843 | HMDB0294452 | 0.00 | 3.37 | ^△^DG(20:4(5Z,7E,11Z,14Z)-OH(9)/0:0/10:0) | Glycerides |  |  |
| 1008 | C_10_H_20_NO_4_ | 218.13847 | 2.914 | 159.0652；85.0288 | HMDB0240784 | 0.05 | 1.50 | ^△^O-Propanoyl-D-carnitine | Acylcarnitines |  |  |

Note：#Secondary fragments, ^△^Website search (https://www.mzcloud.org; <https://www.chemspider.com).> H-TSA vs. Model Control. The fold change (FC) is calculated as H-TSA / MC.

# Table S3

**Table S3 Oral glucose tolerance test (OGTT) and gavage scheme in db/db mice**

| **Group** | **Mice (8 week, n=5)** | **Dosage（Gavage, 200μL）** | **Oral glucose tolerance test** |  |  |
| --- | --- | --- | --- | --- | --- |
| NC | db/m mice | First, starch (2g/kg body weight); then, pure water | 0 min,30 min,60 min,120 min (1~2 μL, a blood glucose meter and test strips (Sinocare/Sanno)) |  |  |
| MC | db/db mice | First, starch (2g/kg body weight); then, pure water |  |  |  |
| AK | db/db mice | First, starch (2g/kg body weight); then, 50mg/kg |  |  | Carbon dioxide euthanasia |
| L-TSA | db/db mice | First, starch (2g/kg body weight); then, 50mg/kg |  |  |  |
| H-TSA | db/db mice | First, starch (2g/kg body weight); then, 100mg/kg |  | 180 min (~100 µL),300 min (~100 µL) |  |
|  |  |  |  |  |  |
| Table S4 **Table S4 Sampling scheme for serum and intestinal content analyses in db/db mice** | | | | | |
|  | **Mice (8 week, n=4)** |  | **Insulin, GLP-1, α-Glucosidase** |  |  |
| MC | db/db mice | First, starch (2g/kg body weight); then, pure water | 0 min (~100 µL),30 min (~900 µL) |  | Cervical dislocation |
| H-TSA | db/db mice | First, starch (2g/kg body weight); then, 100mg/kg | 0 min (~100 µL),30 min (~900 µL) |  |  |

Note：NC, Normal control group; MC, Model control group; AK, Acarbose; L-TSA, Low dose TSA group; H-TSA, High-dose TSA group.

# Fig S1 Tea processing flowchart.


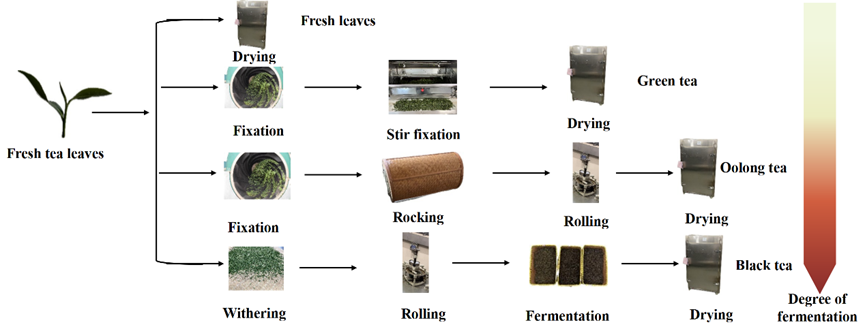


# Fig S2 Total ion chromatogram of tea infusion; A-Fresh leaves(n=3); B-Green tea; C-Oolong tea; D-Black tea.


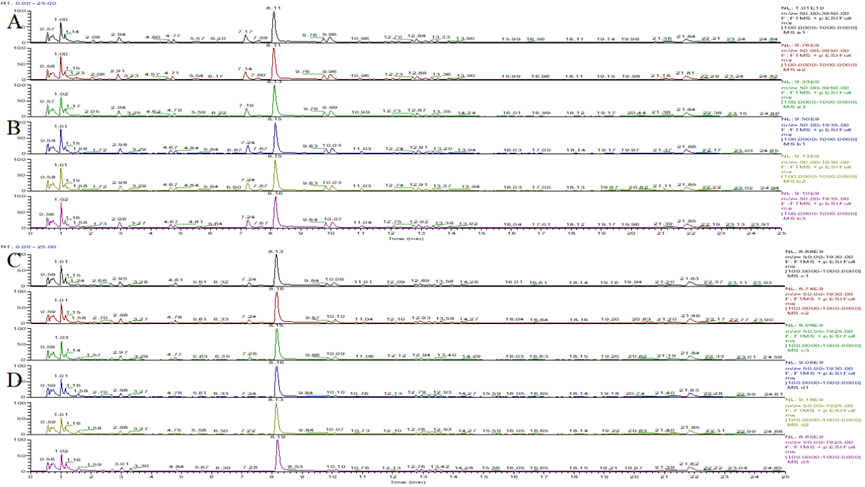

Supplement: Supplementary file 1 — Supplementary material [file mmc1.docx]
